# Mortality Associated With Influenza and Respiratory Syncytial Virus in the US, 1999-2018

**DOI:** 10.1001/jamanetworkopen.2022.0527

**Published:** 2022-02-28

**Authors:** Chelsea L. Hansen, Sandra S. Chaves, Clarisse Demont, Cécile Viboud

**Affiliations:** 1 Division of International Epidemiology and Population Studies, Fogarty International Center, National Institutes of Health, Bethesda, Maryland; 2 Brotman Baty Institute for Precision Medicine, University of Washington School of Medicine, Seattle; 3 Department of Modeling, Epidemiology and Data Science, Sanofi Pasteur, Lyon, France; 4 Foundation for Influenza Epidemiology, Fondation de France, Paris, France; 5 Global RSV Medical Franchise Department, Sanofi Pasteur, Lyon, France

## Abstract

**Importance:**

Respiratory syncytial virus (RSV) mortality estimates have not been updated since 2009, and no study has assessed changes in influenza mortality after the 2009 pandemic. Updated burden estimates are needed to characterize long-term changes in the epidemiology of these viruses.

**Objective:**

To evaluate excess mortality from RSV and influenza in the US from 1999 to 2018.

**Design, Setting, and Participants:**

This cross-sectional study used data from 50.3 million US death certificates from 1999 to 2018 to create age-specific linear regression models and assess weekly mortality fluctuations above a seasonal baseline associated with RSV and influenza. Statistical analysis was performed for 1043 weeks from January 3, 1999, to December 29, 2018.

**Main Outcomes and Measures:**

Excess mortality associated with RSV and influenza estimated from the difference between observed and expected underlying respiratory mortality each season.

**Results:**

There were 50.3 million death certificates (50.1% women and 49.9% men; mean [SD] age at death, 72.7 [18.6] years) included in this analysis, 1.0% for children younger than 1 year and 73.4% for adults aged 65 years or older. A mean of 6549 (95% CI, 6140-6958) underlying respiratory deaths were associated with RSV annually, including 96 (95% CI, 92-99) deaths among children younger than 1 year. For influenza, there were 10 171 (95% CI, 9652-10 691) underlying respiratory deaths per year, with 23 deaths (95% CI, 19-27) among children younger than 1 year. The highest mean mortality rate per 100 000 population for both viruses was among adults aged 65 years or older at 14.7 (95% CI, 13.8-15.5) for RSV and 20.5 (95% CI, 19.4-21.5) for influenza. A lower proportion of influenza deaths occurred among those aged 65 years or older compared with earlier estimates (75.1% [95% CI, 67.4%-82.8%]). Influenza mortality was highest among those aged 65 years or older in seasons when A/H3N2 predominated (18 739 [95% CI, 16 616-21 336] deaths in 2017-2018) and among those aged 5 to 49 years when A/H1N1pdm2009 predominated (1683 [95% CI, 1583-1787] deaths in 2013-2014). Results were sensitive to the choice of mortality outcome and method, with the broadest outcome associated with annual means of 23 352 (95% CI, 21 814-24 891) excess deaths for RSV and 27 171 (95% CI, 25 142-29 199) for influenza.

**Conclusions and Relevance:**

This study suggests that RSV poses a greater risk than influenza to infants, while both are associated with substantial mortality among elderly individuals. Influenza has large interannual variability, affecting different age groups depending on the circulating virus. The emergence of the influenza A/H1N1pdm2009 pandemic virus in 2009 shifted mortality toward middle-aged adults, a trend still observed to date. This study’s estimates provide a benchmark to evaluate the mortality benefits associated with interventions against respiratory viruses, including new or improved immunization strategies.

## Key Points

**Question:**

What was the excess mortality from respiratory syncytial virus (RSV) and influenza in the US from 1999 to 2018?

**Findings:**

This cross-sectional study estimates a mean of 6549 underlying respiratory deaths associated with RSV each year (range, 5035-7645) and estimates a mean of 10 171 underlying respiratory deaths associated with influenza per year (range, 393 to 23 176), with greater interannual variation for influenza than for RSV. The highest mortality for both viruses was among individuals aged 65 years or older; RSV mortality was 5-fold higher than influenza mortality among children younger than 1 year.

**Meaning:**

This study suggests that, despite changes in epidemiology, endemic respiratory viruses continue to have a significant death toll in the US, especially among infants and elderly individuals.

## Teaser

This cross-sectional study uses data from 50.3 million US death certificates to evaluate excess mortality from respiratory syncytial virus (RSV) and influenza from 1999 to 2018.

## Introduction

Historically, influenza viruses and respiratory syncytial virus (RSV) have been associated with substantial mortality among young children and elderly individuals.^1^ Time-series modeling of vital statistics data is one of the key approaches used to estimate the excess mortality burden of RSV and influenza. In this approach, week-to-week fluctuations in mortality above a seasonal baseline are associated with specific respiratory pathogens based on regression models. In the US, the latest RSV excess mortality estimates span the 1990-1999 and 1997-2009 time periods,^1,2^ and no study has assessed long-term changes in influenza excess mortality before and after the 2009 pandemic, to our knowledge. Furthermore, the last 2 decades have seen substantial changes in demographic characteristics, health care access, and patient management and increased influenza vaccination coverage in pediatric populations.^3,4,5,6,7,8,9,10,11^

We assessed RSV-related excess mortality trends from 1999 to 2018 compared with excess mortality from influenza and explored statistical refinements to existing approaches. Updated burden estimates are needed to characterize the epidemiology of these viruses and document changes from decade to decade, providing perspective on public health interventions contrasted with secular trends. These estimates can also serve as a benchmark to measure changes in the circulation of influenza viruses and RSV during and after the SARS-CoV-2 pandemic.

## Methods

### Mortality Data

In this cross-sectional study, we requested death certificates from the National Center for Health Statistics from 1999 to 2018. We used underlying cause of death categorized based on the *International Statistical Classification of Diseases and Related Health Problems, Tenth Revision*^12^ as pneumonia and influenza (UPI: codes J09-J18), respiratory (UR: codes J00-J99), respiratory and circulatory (URC: codes I00-J99), and all causes. Our main analysis focused on the UR category because it includes bronchiolitis deaths and may be more appropriate for estimating RSV than UPI deaths, especially for infants. Deaths were aggregated by week, age group (<1, 1-4, 5-49, 50-64, and ≥65 years), and US Department of Health and Human Services region (excluding US territories, Alaska, and Hawaii). Midyear population estimates by age group and US Department of Health and Human Services region were obtained from the Centers for Disease Control and Prevention and the National Center for Health Statistics.^13^ This research was based on modeling of public use aggregated data that do not include identifiable personal information and was exempt from institutional review board review and approval per the Common Rule that covers public health surveillance studies for disease trends. This study followed the Strengthening the Reporting of Observational Studies in Epidemiology (STROBE) reporting guideline for cross-sectional studies.

### Viral Surveillance Data

We obtained weekly regional data on influenza testing and outpatient visits for influenza-like illness from the “cdcfluview” R package.^14^ We calculated a regional proxy for influenza virus circulation by multiplying the weekly proportion of influenza positive tests by the weekly percentage of influenza-like illnesses.^15,16^ In the main analyses, we used all influenza subtypes combined as a proxy for influenza incidence, but we also ran sensitivity analyses using subtype-specific proxies on weekly and seasonal time scales.^17^ An additional description is provided in the eMethods in the Supplement. Weekly regional RSV data were obtained from the National Respiratory and Enteric Virus Surveillance System.^18^ We used the weekly proportion of positive RSV test results as a proxy for RSV circulation. Information on RSV test types (polymerase chain reaction [PCR], antigen, or viral culture) was available starting in 2010. We defined seasons from epidemiologic week 27 to epidemiologic week 26 of the following year for both RSV and influenza.^19^ For weeks when no specimens were collected, the proportion of positive test results was set to zero.

### Statistical Analysis

Statistical analysis was performed for 1043 weeks from January 3, 1999, to December 29, 2018. To estimate excess mortality for each selected cause of death, we fit linear regression models by week and age, following: *mr*_(*t*; *c*, *a*) = β_0_ + *ns*(*t*) + ∑^19^*_s_* _= 1_ β_1,_ *_s_* × L1(flu)(*t*) + β_2_ × L2(RSV)(*t*), where *mr_*(*t*; *c*, *a*) represents the 5-week moving mean of the mortality rate per 100 000 population for cause *c*, age group *a*, and week *t*; *ns*(*t*) is a natural cubic spline, with 60 *df* representing a smooth function of time for seasonality in mortality that is not associated with influenza or RSV, and *s*1 to *s*19 account for between-season variability in influenza severity; and flu(*t*) and RSV(*t*) are the weekly influenza and RSV circulation proxies, respectively. For the national model, we used the weekly mean of the regional influenza and RSV proxies (same for all ages), weighted by regional populations. As part of model calibration, we allowed for a lag of up to 8 weeks between viral activity and mortality, separately for influenza and RSV (represented by L1 and L2, respectively). The Akaike information criterion was used to select the optimal virus lag for UR mortality models. The same lag was applied to the other causes of death (UPI, URC, and all-cause mortality), but we let each age group have a different lag (eMethods in the Supplement). The same model was then used for regional estimates.

Seasonal influenza and RSV excess mortality rates were estimated as the weekly viral surveillance covariate multiplied by the viral coefficient and summed for all weeks of a given respiratory illness season. We used 1-way analysis of variance to test for differences in seasonal excess mortality between regions and the Tukey method for pairwise comparisons. Residual bootstrapping (1000 replicates) was used to calculate 95% CIs.

We ran sensitivity analyses to examine the association of increased PCR testing for RSV over time^19^ and to assess the robustness of regional-level mortality estimates (eMethods in the Supplement). Statistical analyses were performed in R, version 4.1.0 (R Group for Statistical Computing).^20^ We considered *P* < .05 to be statistically significant. All hypothesis tests were 2-sided.

## Results

### Viral Surveillance and Underlying Causes of Death

**Figure 1.**
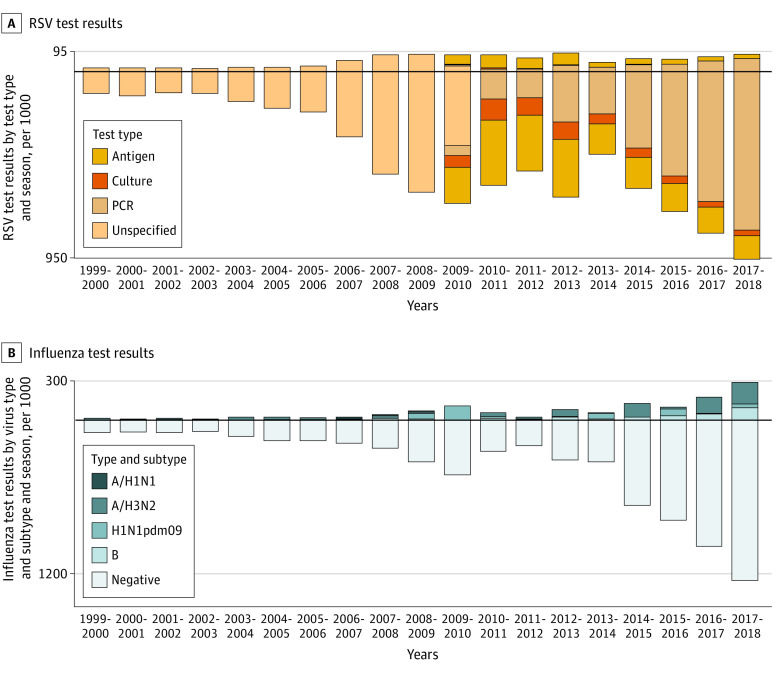
Respiratory Syncytial Virus (RSV) Results by Test Type and Season and Influenza Results by Type, Subtype, and Season A, RSV test results. Bars indicate the number of RSV tests conducted each season from 1999-2000 to 2017-2018, with bars above the horizontal line corresponding to the number of positive test results and bars below the line corresponding to the number of negative test results. Bar colors indicate the type of test used (antigen, culture, or polymerase chain reaction [PCR]), where those data are available beginning in 2010. B, Influenza test results. Bars indicate the number of influenza tests conducted each season from 1999-2000 to 2017-2018, with bars above the horizontal line corresponding to the number of positive test results and bars below the line corresponding to the number of negative test results. Bar colors above the horizontal line (positive test results) indicate the influenza type and subtype detected.

**Figure 2.**
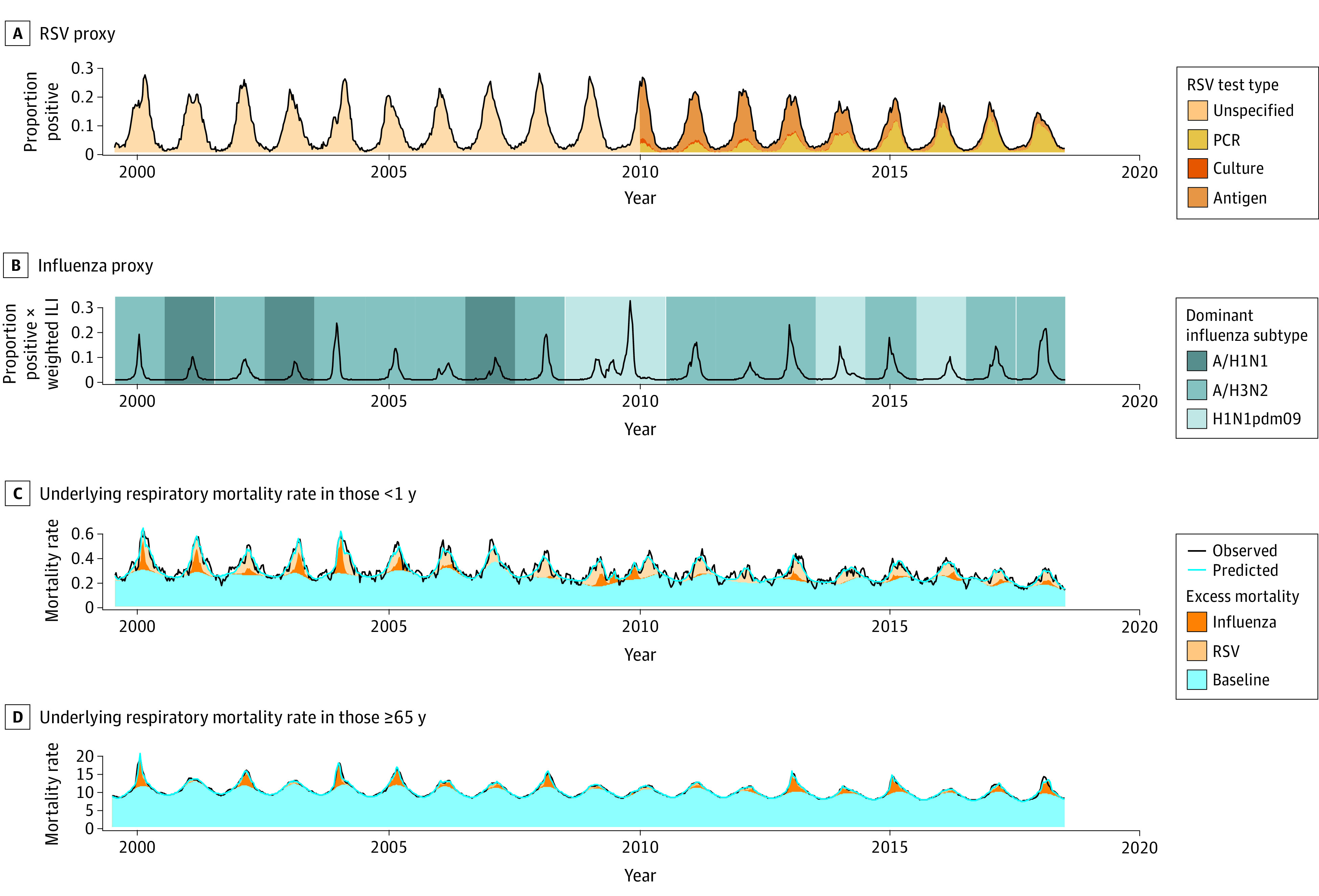
Weekly Time Series for Respiratory Syncytial Virus (RSV) and Influenza Surveillance Proxies and the Underlying Respiratory Mortality Rate per 100 000 Population in Children Younger Than 1 Year and Adults Aged 65 Years or Older A, Weekly time series of the proportion of RSV tests with positive results. The different shades of color indicate the type of test used. B, Weekly time series of the proportion of influenza tests with positive results multiplied by weekly, weighted influenza-like illness (ILI) outpatient visits. The different shades of color indicate the dominant influenza A subtype circulating during each season. C, Weekly time series of the underlying respiratory mortality rate for children younger than 1 year (black line), the weekly underlying respiratory mortality rate for children younger than 1 year estimated by the model (blue line), the estimated weekly baseline mortality for children younger than 1 year (blue area), the weekly estimated excess mortality for children younger than 1 year associated by the model with influenza (brown area), and the weekly excess mortality for children younger than 1 year associated by the model with RSV (tan area). D, Same as panel C but for adults aged 65 years or older. PCR indicates polymerase chain reaction.

Respiratory surveillance testing increased during the study period by approximately 8-fold for RSV and 14-fold for influenza (Figure 1; eTable 1 in the Supplement). Polymerase chain reaction testing for RSV surpassed antigen testing in the 2013-2014 season, reaching 83.6% of all tests in 2017-2018 (Figure 1A; eTable 1 in the Supplement). The mean seasonal peak in the percentage of positive results for RSV samples decreased from 23.6% in the years prior to 2010 to 19.4% in the years afterward (Figure 2A; *P* < .003). Influenza circulation showed more season-to-season variability than RSV, with the most intense season corresponding to the 2009-2010 A/H1N1pdm2009 pandemic (Figure 2B). Influenza A/H3N2 predominated in 12 of the 19 studied seasons, whereas seasonal A/H1N1 predominated in 3 seasons and A/H1N1pdm2009 predominated in 4 seasons.

There were 50.3 million death certificates (50.1% women and 49.9% men; mean [SD] age at death, 72.7 [18.6] years) included in this analysis. Children younger than 1 year represented 1.0% of the data, while adults aged 65 years or older represented 73.4%. The annual mortality rate for all death categories decreased in the initial years of the study and rebounded in the later years (eTable 2 and eFigure in the Supplement). There was a significant linear decrease in the age-specific mortality rates for children younger than 1 year and adults aged 65 years or older for all death categories (eFigure in the Supplement). Similarly, winter peaks in UR mortality also decreased from 0.6 to 0.3 (*P* < .001) among children younger than 1 year and from 18.9 to 13.9 (*P* = .01) among adults aged 65 years or older (Figure 2C and D).

### Excess RSV Mortality Estimates

**Figure 3.**
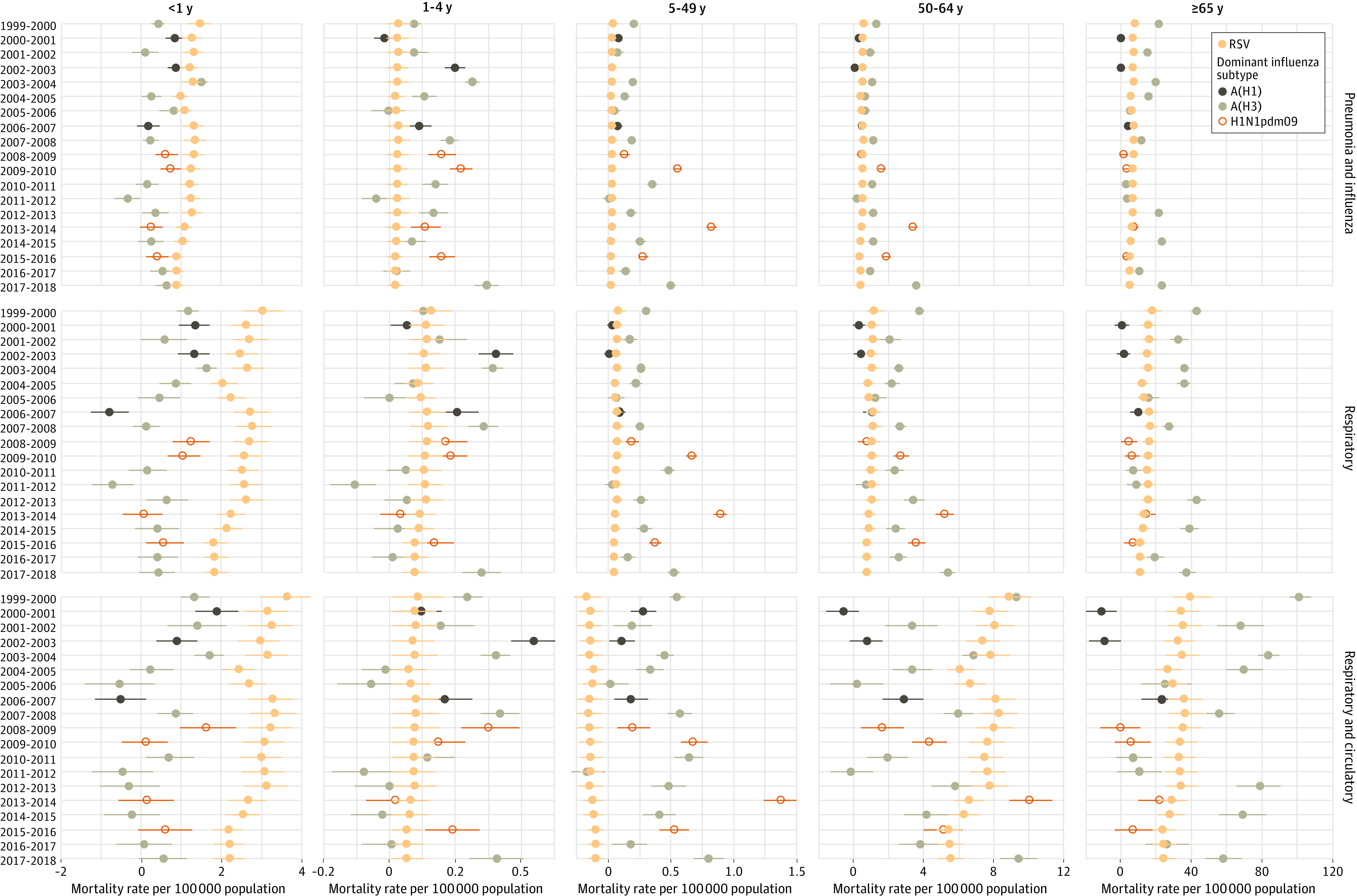
Estimated Excess Mortality Rate for 3 Underlying Causes of Death for Respiratory Syncytial Virus (RSV) and Influenza, by Season and by Age Group Point estimates and 95% CIs for RSV and influenza-associated excess mortality rates per 100 000 population for each respiratory virus season (from 1999-2000 to 2017-2018), age group (<1, 1-4, 5-49, 50-64, and ≥65 years), and underlying cause of death (pneumonia and influenza, respiratory, and respiratory and circulatory). Yellow points indicate RSV mortality, dark gray points indicate influenza A/H1N1 mortality, light gray points indicate influenza A/H3N2 mortality, and open points indicate influenza A/H1N1pdm2009 mortality.

There was a mean of 6549 (95% CI, 6140-6958) annual excess UR deaths associated with RSV, ranging from 5035 to 7645 depending on the season (Table; eTable 3 in the Supplement). The highest mean, RSV-associated UR mortality rate was among adults aged 65 years or older at 14.7 per 100 000 (95% CI, 13.8-15.5 per 100 000), followed by children younger than 1 year at 2.4 per 100 000 (95% CI, 2.3-2.5 per 100 000) (Table). For infants, this rate corresponds to a mean of 96 (95% CI, 92-99) annual RSV-associated UR deaths. The lowest mean excess mortality rates for RSV were among those aged 1 to 4 years and those aged 5 to 49 years (Figure 3; eTable 3 in the Supplement). Including underlying circulatory deaths increased the estimated mean RSV-associated mortality rate in age groups older than 50 years (particularly those aged 50-64 years) but not in younger age groups. Children younger than 1 year also had a mean of 47 (95% CI, 45-49) annual RSV-associated UPI deaths and a mean of 115 (95% CI, 110-120) annual RSV-associated URC deaths. Adults aged 65 years or older had a mean of 2655 (95% CI, 2506-2804) annual RSV-associated UPI deaths, a mean of 5800 (95% CI, 5461-6139) annual RSV-associated UR deaths, and a mean of 12 604 (95% CI, 11 808-13 399) annual RSV-associated URC deaths. Sensitivity analyses using models based on all-cause deaths—the broadest mortality outcome available—resulted in 23 352 (95% CI, 21 814-24 891) all-age excess deaths for RSV (Table).

**Table.** Estimated Mean, Annual Age-Specific Influenza and RSV Deaths and Mortality Rates per 100 000 Population, 1999-2000 to 2017-2018, US

Underlying cause of death and age group, y	RSV deaths, No. (95% CI)	RSV mortality rate per 100 000 population (95% CI)	Influenza deaths, No. (95% CI)	Influenza mortality rate per 100 000 population (95% CI)
Pneumonia and influenza				
<1	47 (45 to 49)	1.2 (1.1 to 1.2)	18 (16 to 21)	0.5 (0.4 to 0.5)
1-4	5 (3 to 6)	0.0 (0.0 to 0.0)	23 (21 to 25)	0.1 (0.1 to 0.2)
5-49	59 (46 to 72)	0.0 (0.0 to 0.0)	419 (403 to 436)	0.2 (0.2 to 0.2)
50-64	250 (229 to 272)	0.5 (0.4 to 0.5)	635 (606 to 664)	1.1 (1.1 to 1.2)
≥65	2655 (2506 to 2804)	6.7 (6.3 to 7.1)	4168 (3968 to 4367)	10.2 (9.7 to 10.7)
Total	3016 (2829 to 3203)	1.0 (0.9 to 1.1)	5263 (5014 to 5512)	1.7 (1.7 to 1.8)
Respiratory				
<1	96 (92 to 99)	2.4 (2.3 to 2.5)	23 (19 to 27)	0.6 (0.5 to 0.7)
1-4	20 (18 to 22)	0.1 (0.1 to 0.1)	24 (21 to 27)	0.2 (0.1 to 0.2)
5-49	124 (108 to 141)	0.1 (0.1 to 0.1)	519 (497 to 541)	0.3 (0.3 to 0.3)
50-64	508 (460 to 556)	1.0 (0.9 to 1.0)	1322 (1260 to 1384)	2.4 (2.2 to 2.5)
≥65	5800 (5461 to 6139)	14.7 (13.8 to 15.5)	8284 (7855 to 8713)	20.5 (19.4 to 21.5)
Total	6549 (6140 to 6958)	2.2 (2.0 to 2.3)	10 171 (9652 to 10 691)	3.4 (3.2 to 3.5)
Respiratory and circulatory				
<1	115 (110 to 120)	2.9 (2.8 to 3.0)	21 (15 to 27)	0.5 (0.4 to 0.7)
1-4	14 (11 to 17)	0.1 (0.1 to 0.1)	28 (24 to 31)	0.2 (0.2 to 0.2)
5-49	−248 (−285 to −211)	−0.1 (−0.2 to −0.1)	771 (719 to 823)	0.4 (0.4 to 0.4)
50-64	3868 (3749 to 3987)	7.2 (7.0 to 7.5)	2265 (2120 to 2410)	4.1 (3.8 to 4.4)
≥65	12 604 (11 808 to 13 399)	31.9 (29.8 to 33.9)	14 496 (13 465 to 15 528)	36.4 (33.9 to 38.8)
Total	16 352 (15 393 to 17 311)	5.4 (5.0 to 5.8)	17 581 (16 343 to 18 819)	5.8 (5.4 to 6.2)
All causes				
<1	106 (82 to 131)	2.7 (2.1 to 3.3)	81 (52 to 110)	2.1 (1.3 to 2.8)
1-4	168 (157 to 179)	1.1 (1.0 to 1.1)	51 (37 to 64)	0.3 (0.2 to 0.4)
5-49	−1767 (−1903 to −1631)	−0.9 (−1.0 to −0.9)	1831 (1648 to 2014)	1.0 (0.9 to 1.1)
50-64	6327 (6138 to 6516)	11.8 (11.5 to 12.2)	3543 (3291 to 3795)	6.4 (6.0 to 6.9)
≥65	18 518 (17 340 to 19 695)	46.8 (43.8 to 49.8)	21 665 (20 115 to 23 215)	54.2 (50.4 to 58.0)
Total	23 352 (21 814 to 24 891)	7.8 (7.1 to 8.4)	27 171 (25 142 to 29 199)	9.0 (8.3 to 9.7)

Abbreviation: RSV, respiratory syncytial virus.

Using the proportion of positive results from only antigen tests from 2010 onward increased RSV-associated UR mortality between 2010-2011 and 2017-2018 by a mean of 20 (95% CI, 10-30) deaths per year among children younger than 1 year and 2400 (95% CI, 1700-3000) deaths per year among adults aged 65 years or older (eTable 4 in the Supplement).

### Excess Influenza Mortality Estimates

The mean number of annual UR deaths associated with influenza was 10 171 (95% CI, 9652-10 691), ranging from 393 in 2000-2001 to 23 176 in 2017-2018, the worst season during our study period (Table; eTable 5 in the Supplement). There was more season-to-season variability in the estimated excess mortality rate associated with influenza than with RSV (Figure 3; eTable 5 in the Supplement). The highest mean, excess, UR influenza mortality rate was among adults aged 65 years or older at 20.5 per 100 000 (95% CI, 19.4-21.5 per 100 000), followed by adults aged 50 to 64 years at 2.4 per 100 000 (95% CI, 2.2-2.5 per 100 000) (Table). The mean number of annual influenza-associated UR deaths among children younger than 1 year was 23 (95% CI, 19-27), with a maximum of 70 (95% CI, 50-80) excess deaths in 2003-2004 (eTable 5 in the Supplement). The lowest influenza mortality rates were among children 1 to 4 years of age and those aged 5 to 49 years. The only age group that had a consistently lower mortality from influenza than from RSV was children younger than 1 year (Figure 3). Linear trends in influenza-associated UR mortality rates increased significantly for those aged 50 to 64 years. Sensitivity analyses considering broader mortality outcomes resulted in substantially higher burden estimates, with 27 171 (95% CI, 25 142-29 199) annual excess deaths associated with influenza, on average, based on all-cause mortality.

A lower proportion of influenza deaths occurred among those aged 65 years or older compared with earlier estimates (75.1% [95% CI, 67.4%-82.8%]). For adults aged 65 years or older, the highest influenza mortality rates for all death categories occurred during seasons dominated by A/H3N2 circulation (Figure 3), with 18 510 (95% CI, 16 151-20 722) influenza-associated UR deaths in 2012-2013 and 18 739 [95% CI, 16 616-21 336) influenza-associated UR deaths in 2017-2018 (eTable 5 in the Supplement). The highest influenza mortality rates for those aged 5 to 49 years occurred during seasons when A/H1N1pdm2009 predominated, with an estimated 1683 (95% CI, 1583-1787) UR deaths in 2013-2014. Influenza A/H3N2 was associated with a mean of 7816 (95% CI, 7092-8537) UR deaths per year, the most of any subtype (eTable 6 in the Supplement). Influenza B was associated with a mean of 2300 (95% CI, 1439-3163) deaths per year, influenza A/H1N1pdm2009 was associated with a mean of 772 (95% CI, 267-1273) deaths per year, and seasonal influenza A/H1N1 was associated with negative estimates except among children. Influenza B did not predominate in any season included in our study but was present in all seasons, and thus it was associated with more deaths, on average, than influenza A/H1N1pdm2009 or influenza A/H1N1, which circulated for only a portion of the study period. The relative importance of influenza A/H1N1pdm2009 and influenza B differed by age, with a larger influenza B burden for adults older than 50 years and a larger influenza A/H1N1pdm2009 burden for those younger than 50 years.

### Regional Excess Mortality for Those Aged 65 Years or Older

**Figure 4.**
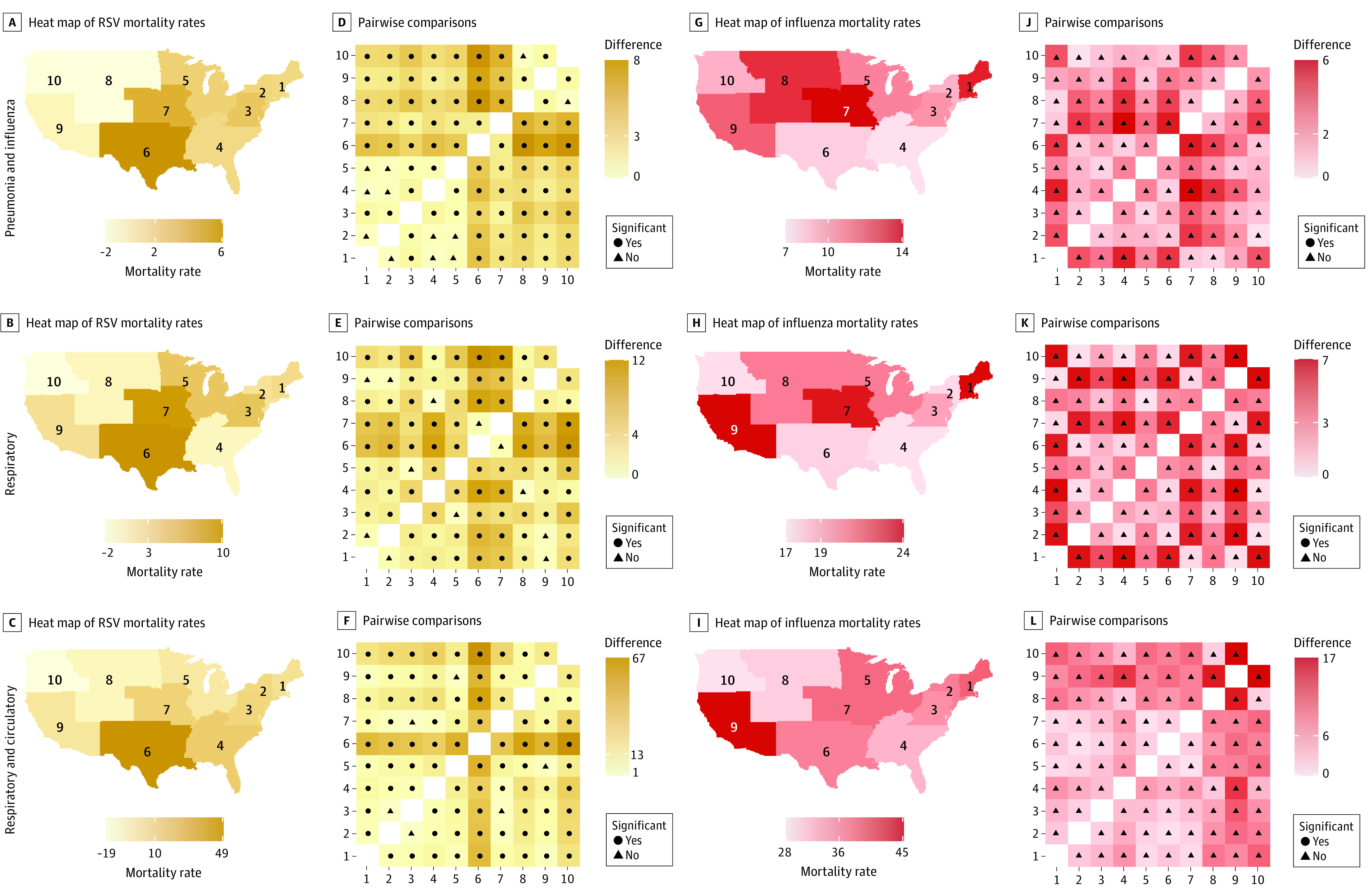
Estimated Mean Annual Excess Influenza and Respiratory Syncytial Virus (RSV) Mortality in Adults Aged 65 Years or Older for 3 Underlying Causes of Death, by US Department of Health and Human Services (HHS) Region and Pairwise Comparisons Between HHS Regions A-C, Heat maps show estimated mean, annual excess underlying mortality rates associated with RSV for each HHS region, with lighter shades indicating lower RSV mortality rates and darker shades, higher RSV mortality rates. Numbers on color scale are minimum, median, and maximum RSV mortality rates. Numbers on map indicate HHS regions. Panels G-I show these data for mortality rates associated with influenza. D-F, Pairwise comparisons of mean annual excess underlying RSV mortality rates between HHS regions (1-10), with lighter shades indicating smaller absolute difference between mean values and darker shades, larger absolute difference between mean values. Numbers on color scale indicate minimum, median, and maximum difference in mean values. Black points in center of tiles indicate statistically significant differences, whereas triangles indicate differences that are not statistically significant. Panels J-L show these data for mortality rates associated with influenza. All rates are per 100 000.

Consistent with national analyses, season-to-season variability in regional estimates was higher for influenza than RSV. The mean mortality rate among those aged 65 years or older was higher for influenza than for RSV for all regions and categories of death (Figure 4). In pairwise comparisons, most regions had significantly different mean, excess RSV mortality rates, with adjoining regions and coasts being more similar. The highest mean, excess RSV UR mortality rate in individuals older than 65 years was in region 6 at 10.1 per 100 000 (95% CI, 9.0-11.2 per 100 000), while regions 8 and 10 had negative estimates, with the lowest estimate found in region 10 at –1.6 per 100 000 (95% CI, –2.4 to –0.9 per 100 000). There were no significant differences between regions in excess influenza mortality rates.

## Discussion

We estimated age- and region-specific excess mortality rates for RSV and influenza in 19 recent seasons in the US in the context of gradually decreasing all-cause mortality in the youngest and oldest populations. We estimated that 6549 UR deaths were associated with RSV annually, ranging from 5035 to 7645. Influenza was associated with 10 171 UR deaths per year, ranging from 393 to 23 176. Estimates based on all-cause mortality were nearly 3-fold higher. Influenza-related mortality was characterized by large interannual fluctuations, with high rates among elderly individuals, especially when influenza A/H3N2 predominated. Since the 2009 pandemic, influenza-related mortality rates have increased among those aged 5 to 49 years and among those aged 50 to 64 years, likely associated with the circulation of the influenza A/H1N1pdm2009 virus. Respiratory syncytial virus showed less variability in annual mortality rates by season but had marked geographical variation among those aged 65 years or older, which we did not see for influenza. Although the highest mortality rate from RSV was among older adults, the excess UR mortality rate among those younger than 1 year was 5-fold higher for RSV than for influenza. Our study shows that, despite changes in epidemiology, endemic respiratory viruses continue to have a significant death toll in the US.

Our RSV estimates can be compared with the seminal study by Thompson et al^1^ that applies a similar excess mortality approach to 1990-1999. For children younger than 1 year, the authors estimated the annual mean RSV-associated deaths to be 124 for UPI and 211 for URC.^1^ These estimates are about twice our estimates 20 years later at 47 (95% CI, 45-49) for UPI and 115 (95% CI, 110-120) for URC (Table). Increased palivizumab use for at-risk infants could play a role in these differences.^21^ Furthermore, mortality for all categories has decreased substantially among infants in the last 20 years in the US, likely affecting mortality associated with winter respiratory pathogens. For adults aged 65 years or older, Thompson et al^1^ estimated the annual mean RSV-associated deaths to be 2388 for UPI and 8811 for URC, lower than our estimates of 2655 (95% CI, 2506-2804) excess UPI deaths and 12 604 (95% CI, 11 808-13 399) excess URC deaths (Table). Accounting for population growth between the 2 study periods reduces these differences. For the period from 1997 to 2009, Matias et al^2^ estimates 11 300 respiratory deaths for all ages. This estimate is higher than our estimate, but the mortality outcome includes causes of death listed anywhere on the death certificate rather than restricting analysis to the underlying cause of death.

Our influenza mortality estimates for children aged 1 to 4 years were half the earlier estimates by Thompson et al^1^ for 1979-1999 (URC category) but similar for children younger than 1 year. After the 2003-2004 influenza season,^22^ influenza vaccination was recommended for children aged 6 to 23 months.^23^ Influenza vaccination has increased in this age group from 7.4% receiving at least 1 dose in 2002-2003^8^ to between 61% and 67% since 2010-2011.^11^ Although seasonal influenza mortality estimates did not decrease linearly for those aged 1 to 4 years, likely owing to the year-to-year variability of influenza seasons, the reported changes in influenza-related excess mortality for this age group may reflect the benefit associated with vaccination.

Our estimated influenza deaths among adults aged 65 years or older are less than half the 7326 annual UPI deaths and the 32 651 annual URC deaths presented by Thompson et al^1^ from 1979 to 1999. We believe these differences reflect true changes in the epidemiology of influenza. Whereas 90% of influenza deaths in previous estimates were among adults aged 65 years or older,^1,24,25^ this age group made up 75.1% (95% CI, 67.4%-82.8%) of influenza deaths in our analysis. Although vaccination coverage was stable during the study period in this age group,^26^ the use of a high-dose influenza vaccine, licensed in 2009, has increased over time and has been shown to provide improved protection.^27,28,29^ Also, circulation of influenza A/H1N1pdm2009 in the post-2009 pandemic period has resulted in relatively mild influenza seasons for those aged 65 years or older, possibly owing to protection from early-life exposures to influenza A/H1N1.^30,31^

In comparing our influenza mortality estimates with those of previously published studies that overlap with the period of our analysis, we found that the choice of method accounts for much of the difference between studies (eTable 7 in the Supplement).^32,33^ For the 1999-2007 period, our estimates are similar to those presented by Goldstein et al^16^ and Quandelacey et al,^34^ which use a similar modeling approach to ours. We also found that the choice of mortality outcome was associated with excess mortality estimates, with broader categories resulting in higher estimated deaths, such as those seen in the estimates by Matias et al^2^ and Rolfes et al.^35^ Including associated causes of death listed anywhere in the death certificate increased our estimates by 40% to 100%.

To provide timely estimates of deaths during the 2009 influenza A/H1N1pdm2009 pandemic, Shrestha et al^30^ developed a multiplier approach that extrapolates influenza mortality from laboratory-confirmed, in-hospital influenza deaths. Deaths are then inflated based on a proportion estimate of deaths occurring outside hospital settings. Although direct comparison with this method is difficult, this approach is perhaps better able to ascertain the full spectrum of deaths associated with influenza and hence is more comparable to all-cause excess mortality. Our influenza excess mortality estimates for all-cause mortality are similar to those using a multiplier approach, especially during severe seasons (eTable 7 in the Supplement).^36^ Although all-cause mortality is usually considered a poorly specific outcome for studies of respiratory virus mortality, using respiratory and circulatory deaths listed as associated causes captured approximately 90% of the influenza burden estimated using all-cause mortality and may be a good compromise between sensitivity and specificity.

Our results support previous work suggesting that RSV may pose a greater risk than influenza to older adults in seasons when influenza A/H1 predominates.^37,38^ Despite RSV’s substantial association with mortality among older adults, 1 survey found that less than half of physicians ordered RSV testing for community-dwelling older adults with acute respiratory infection.^39^ A better understanding of RSV testing practices is an important area for future work given recent changes and would provide further evidence to support recommendations for an RSV vaccine and monoclonal antibody products soon to be licensed. Although data were too sparse to allow for regional excess mortality estimates for children younger than 1 year, understanding regional differences in RSV circulation among infants will be important for future immunization programs because implementation may consider different exposure periods.^40,41,42^ These differences remain an important area for future RSV research.

Several studies have noted that nonpharmaceutical interventions put in place to mitigate the SARS-CoV-2 pandemic have reduced infection rates with other endemic respiratory pathogens, including RSV and influenza, to historically low levels.^43,44,45^ How this period of suppressed circulation will change the epidemiology of influenza and RSV when interventions are lifted remains to be seen.

### Limitations

This study has some limitations. Our model may be underestimating RSV mortality in recent years owing to changes in case ascertainment.^46^ Use of multipathogen PCR panels increases the RSV testing pool to include individuals unlikely to have RSV, thus decreasing the weekly percentage of positive test results.^19,46^ Our sensitivity analysis showed that there was little difference in mean estimates over the 20-year period. Although we do not have data by test type for influenza, increased use of PCR testing has likely affected influenza surveillance.^47^ We did not have access to age-specific viral surveillance data, and thus the same proxies were used for all age groups. Our model produced negative estimates for RSV-associated URC deaths among those aged 5 to 49 years and for all mortality outcomes for individuals older than 65 years in regions 8 and 10, suggesting that, for low-risk populations, broad death categories may not be ideal for ascertaining RSV excess mortality. Finally, we did not consider sex or race and ethnicity, which may be important for future work.

## Conclusions

Although the RSV mortality rate has decreased since the 1990s among children younger than 1 year, it is still associated with approximately 100 UR deaths annually in this age group, posing a greater risk than influenza to infants. Respiratory syncytial virus is also an important cause of death among those aged 65 years or older; in some seasons, it is associated with more deaths than influenza. Influenza is characterized by large interannual variability and continues to cause large numbers of deaths, affecting age groups differentially depending on the dominant virus circulating. The emergence of the influenza A/H1N1pdm2009 virus in 2009 has shifted mortality toward middle-aged adults. Our estimates can be used to evaluate the completeness of death certificates, encourage testing for RSV in traditionally lower-risk populations, and provide a benchmark to evaluate the mortality benefits associated with interventions against respiratory viruses, including new or improved immunization strategies.

## Notes

Supplement.
eMethods.
**eTable 1.** Specimens Collected and Positive Tests for Respiratory Syncytial Virus and Influenza by Season, 1999/2000 to 2017/2018, USA **eTable 2.** Population in Millions and Total Underlying Deaths for Pneumonia and Influenza (UPI), Respiratory (UR), and Respiratory and Circulatory (URC), and All-Causes by Age Group, 1999/2000 to 2017/2018, USA **eFigure.** Mortality Rates per 100,000 Population by Season, Age Group and Underlying Cause of Death **eTable 3.** Estimated Excess Respiratory Syncytial Virus Deaths and Mortality Rate per 100,000 Population by Season and Age Group, 1999/2000 to 2017/2018, USA **eTable 4.** Estimated Excess Respiratory Syncytial Virus Deaths and Mortality Rate per 100,000 Population by Season and Age Group, 2010/2011 to 2017/2018, USA When Surveillance Data Are Limited to Antigen-Based Testing Starting in 2010 **eTable 5.** Estimated Excess Influenza Deaths and Mortality Rate per 100,000 Population by Season and Age Group, 1999/2000 to 2017/2018, USA **eTable 6.** Estimated Average Annual Age-Specific Influenza Deaths by Type/Subtype, 1999/2000 to 2017/2018, USA **eTable 7.** Comparison With Other Published Estimates of Excess Influenza Mortality Rate for All Ages per 100,000 Population
eReferences.

## References

[1] Thompson WW, Shay DK, Weintraub E, . Mortality associated with influenza and respiratory syncytial virus in the United States. JAMA. 2003;289(2):179-186. doi:10.1001/jama.289.2.179 12517228

[2] Matias G, Taylor R, Haguinet F, Schuck-Paim C, Lustig R, Shinde V. Estimates of mortality attributable to influenza and RSV in the United States during 1997-2009 by influenza type or subtype, age, cause of death, and risk status. Influenza Other Respir Viruses. 2014;8(5):507-515. doi:10.1111/irv.12258 24975705 PMC4181813

[3] Woolf SH, Schoomaker H. Life expectancy and mortality rates in the United States, 1959-2017. JAMA. 2019;322(20):1996-2016. doi:10.1001/jama.2019.16932 31769830 PMC7146991

[4] Stoll BJ, Hansen NI, Bell EF, ; Eunice Kennedy Shriver National Institute of Child Health and Human Development Neonatal Research Network. Trends in care practices, morbidity, and mortality of extremely preterm neonates, 1993-2012. JAMA. 2015;314(10):1039-1051. doi:10.1001/jama.2015.10244 26348753 PMC4787615

[5] Bhatt CB, Beck-Sagué CM. Medicaid expansion and infant mortality in the United States. Am J Public Health. 2018;108(4):565-567. doi:10.2105/AJPH.2017.304218 29346003 PMC5844390

[6] Driscoll AK, Ely DM. Effects of changes in maternal age distribution and maternal age-specific infant mortality rates on infant mortality trends: United States, 2000–2017. Natl Vital Stat Rep. 2020;69(5):1-18. 32600516

[7] Callaghan WM, Macdorman MF, Shapiro-Mendoza CK, Barfield WD. Explaining the recent decrease in US infant mortality rate, 2007-2013. Physiol Behav. 2017;216(1):73.e1-73.e8. doi:10.1016/j.ajog.2016.09.097 27687216 PMC5182176

[8] Santibanez T, Barker L, Santoli J, ; Centers for Disease Control and Prevention (CDC). Childhood influenza-vaccination coverage—United States, 2002-03 influenza season. MMWR Morb Mortal Wkly Rep. 2004;53(37):863-866. 15385916

[9] Santibanez T, Singleton J, Santoli J, Euler G, Bridges C; Centers for Disease Control and Prevention (CDC). Childhood influenza vaccination coverage—United States, 2003-04 influenza season. MMWR Morb Mortal Wkly Rep. 2006;55(4):100-103. 16456526

[10] Santibanez T, Singleton J, Shaw K, ; Centers for Disease Control and Prevention (CDC). Childhood influenza vaccination coverage—United States, 2004-05 influenza season. MMWR Morb Mortal Wkly Rep. 2006;55(39):1062-1065. 17021590

[11] Centers for Disease Control and Prevention. Full and partial flu vaccination coverage in young children, six immunization information system sentinel sites, 2013-14 through 2017-18. Accessed June 3, 2021. https://www.cdc.gov/flu/fluvaxview/full-partial-vaccination-children-2018.htm

[12] World Health Organization. *ICD-10: International Statistical Classification of Diseases and Related Health Problems, Tenth Revision*. 2nd ed. Accessed May 20, 2021. https://apps.who.int/iris/handle/10665/42980

[13] Centers for Disease Control and Prevention. Bridged-race population estimates. Accessed March 2, 2021. https://wonder.cdc.gov/bridged-race-population.html

[14] Rudis B, McGowan C, Chen JJ, et al. cdcfluview: Retrieve flu season data from the United States Centers for Disease Control and Prevention (“CDC”) “FluView” portal. R Package, version 0.9.4. Published 2021. Accessed March 2, 2021. https://cran.r-project.org/package=cdcfluview

[15] Goldstein E, Cobey S, Takahashi S, Miller JC, Lipsitch M. Predicting the epidemic sizes of influenza A/H1N1, A/H3N2, and B: a statistical method. PLoS Med. 2011;8(7):e1001051. doi:10.1371/journal.pmed.1001051 21750666 PMC3130020

[16] Goldstein E, Viboud C, Charu V, Lipsitch M. Improving the estimation of influenza-related mortality over a seasonal baseline. Epidemiology. 2012;23(6):829-838. doi:10.1097/EDE.0b013e31826c2dda 22992574 PMC3516362

[17] Dushoff J, Plotkin JB, Viboud C, Earn DJD, Simonsen L. Mortality due to influenza in the United States—an annualized regression approach using multiple-cause mortality data. Am J Epidemiol. 2006;163(2):181-187. doi:10.1093/aje/kwj024 16319291

[18] Centers for Disease Control and Prevention. The National Respiratory and Enteric Virus Surveillance System (NREVSS). Accessed June 4, 2021. https://www.cdc.gov/surveillance/nrevss/index.html

[19] Midgley CM, Haynes AK, Baumgardner JL, . Determining the seasonality of respiratory syncytial virus in the United States: the impact of increased molecular testing. J Infect Dis. 2017;216(3):345-355. doi:10.1093/infdis/jix275 28859428 PMC5712458

[20] R Core Team. R: a language and environment for statistical computing. Accessed December 28, 2021. https://www.r-project.org/

[21] American Academy of Pediatrics Committee on Infectious Diseases; American Academy of Pediatrics Bronchiolitis Guidelines Committee. Updated guidance for palivizumab prophylaxis among infants and young children at increased risk of hospitalization for respiratory syncytial virus infection. Pediatrics. 2014;134(2):e620-e638. doi:10.1542/peds.2014-1666 25070304

[22] Bhat N, Wright JG, Broder KR, ; Influenza Special Investigations Team. Influenza-associated deaths among children in the United States, 2003-2004. N Engl J Med. 2005;353(24):2559-2567. doi:10.1056/NEJMoa051721 16354892

[23] Harper SA, Fukuda K, Uyeki TM, Cox NJ, Bridges CB; Centers for Disease Control and Prevention (CDC) Advisory Committee on Immunization Practices (ACIP). Prevention and control of influenza: recommendations of the Advisory Committee on Immunization Practices (ACIP). MMWR Recomm Rep. 2004;53(RR-6):1-40. 15163927

[24] Centers for Disease Control and Prevention (CDC). Estimates of deaths associated with seasonal influenza—United States, 1976-2007. MMWR Morb Mortal Wkly Rep. 2010;59(33):1057-1062. 20798667

[25] Lui KJ, Kendal AP. Impact of influenza epidemics on mortality in the United States from October 1972 to May 1985. Am J Public Health. 1987;77(6):712-716. doi:10.2105/AJPH.77.6.712 3578619 PMC1647063

[26] Centers for Disease Control and Prevention. Estimates of influenza vaccination coverage among adults—United States, 2017–18 flu season. Accessed June 3, 2021. https://www.cdc.gov/flu/fluvaxview/coverage-1718estimates.htm

[27] McGrath LJ, Brookhart MA. On-label and off-label use of high-dose influenza vaccine in the United States, 2010-2012. Hum Vaccin Immunother. 2015;11(3):537-544. doi:10.1080/21645515.2015.1011026 25751700 PMC4514225

[28] DiazGranados CA, Dunning AJ, Kimmel M, . Efficacy of high-dose versus standard-dose influenza vaccine in older adults. N Engl J Med. 2014;371(7):635-645. doi:10.1056/NEJMoa1315727 25119609

[29] Shay DK, Chillarige Y, Kelman J, . Comparative effectiveness of high-dose versus standard-dose influenza vaccines among US Medicare beneficiaries in preventing postinfluenza deaths during 2012-2013 and 2013-2014. J Infect Dis. 2017;215(4):510-517. doi:10.1093/infdis/jiw641 28329311

[30] Shrestha SS, Swerdlow DL, Borse RH, . Estimating the burden of 2009 pandemic influenza A (H1N1) in the United States (April 2009-April 2010). Clin Infect Dis. 2011;52(suppl 1):S75-S82. doi:10.1093/cid/ciq012 21342903

[31] Gostic KM, Bridge R, Brady S, Viboud C, Worobey M, Lloyd-Smith JO. Childhood immune imprinting to influenza A shapes birth year-specific risk during seasonal H1N1 and H3N2 epidemics. PLoS Pathog. 2019;15(12):e1008109. doi:10.1371/journal.ppat.1008109 31856206 PMC6922319

[32] Li L, Wong JY, Wu P, . Heterogeneity in estimates of the impact of influenza on population mortality: a systematic review. Am J Epidemiol. 2018;187(2):378-388. doi:10.1093/aje/kwx270 28679157 PMC5860627

[33] Thompson WW, Moore MR, Weintraub E, . Estimating influenza-associated deaths in the United States. Am J Public Health. 2009;99(suppl 2):S225-S230. doi:10.2105/AJPH.2008.151944 19797736 PMC4504370

[34] Quandelacy TM, Viboud C, Charu V, Lipsitch M, Goldstein E. Age- and sex-related risk factors for influenza-associated mortality in the United States between 1997-2007. Am J Epidemiol. 2014;179(2):156-167. doi:10.1093/aje/kwt235 24190951 PMC3873104

[35] Rolfes MA, Foppa IM, Garg S, . Annual estimates of the burden of seasonal influenza in the United States: a tool for strengthening influenza surveillance and preparedness. Influenza Other Respir Viruses. 2018;12(1):132-137. doi:10.1111/irv.12486 29446233 PMC5818346

[36] Centers for Disease Control and Prevention. Past seasons estimated influenza disease burden. Accessed June 7, 2021. https://www.cdc.gov/flu/about/burden/past-seasons.html

[37] Falsey AR, Hennessey PA, Formica MA, Cox C, Walsh EE. Respiratory syncytial virus infection in elderly and high-risk adults. N Engl J Med. 2005;352(17):1749-1759. doi:10.1056/NEJMoa043951 15858184

[38] Ackerson B, Tseng HF, Sy LS, . Severe morbidity and mortality associated with respiratory syncytial virus versus influenza infection in hospitalized older adults. Clin Infect Dis. 2019;69(2):197-203. doi:10.1093/cid/ciy991 30452608 PMC6603263

[39] Allen KE, Beekmann SE, Polgreen P, . Survey of diagnostic testing for respiratory syncytial virus (RSV) in adults: infectious disease physician practices and implications for burden estimates. Diagn Microbiol Infect Dis. 2018;92(3):206-209. doi:10.1016/j.diagmicrobio.2017.12.011 30177420

[40] Cromer D, van Hoek AJ, Newall AT, Pollard AJ, Jit M. Burden of paediatric respiratory syncytial virus disease and potential effect of different immunisation strategies: a modelling and cost-effectiveness analysis for England. Lancet Public Health. 2017;2(8):e367-e374. doi:10.1016/S2468-2667(17)30103-2 28804787 PMC5541134

[41] Hodgson D, Pebody R, Panovska-Griffiths J, Baguelin M, Atkins KE. Evaluating the next generation of RSV intervention strategies: a mathematical modelling study and cost-effectiveness analysis. BMC Med. 2020;18(1):348. doi:10.1186/s12916-020-01802-8 33203423 PMC7672821

[42] Griffin MP, Yuan Y, Takas T, ; Nirsevimab Study Group. Single-dose nirsevimab for prevention of RSV in preterm infants. N Engl J Med. 2020;383(5):415-425. doi:10.1056/NEJMoa1913556 32726528

[43] Olsen SJ, Azziz-Baumgartner E, Budd AP, . Decreased influenza activity during the COVID-19 pandemic—United States, Australia, Chile, and South Africa, 2020. MMWR Morb Mortal Wkly Rep. 2020;69(37):1305-1309. doi:10.15585/mmwr.mm6937a6 32941415 PMC7498167

[44] Baker RE, Park SW, Yang W, Vecchi GA, Metcalf CJE, Grenfell BT. The impact of COVID-19 nonpharmaceutical interventions on the future dynamics of endemic infections. Proc Natl Acad Sci U S A. 2020;117(48):30547-30553. doi:10.1073/pnas.2013182117 33168723 PMC7720203

[45] Gomez GB, Mahé C, Chaves SS. Uncertain effects of the pandemic on respiratory viruses. Science. 2021;372(6546):1043-1044. doi:10.1126/science.abh3986 34083477

[46] Ambrose CS, Steed LL, Brandon M, Frye K, Olajide IR, Thomson G. National and regional modeling of distinct RSV seasonality thresholds for antigen and PCR testing in the United States. J Clin Virol. 2019;120(June):68-77. doi:10.1016/j.jcv.2019.09.010 31590113

[47] Millman AJ, Reed C, Kirley PD, . Improving accuracy of influenza-associated hospitalization rate estimates. Emerg Infect Dis. 2015;21(9):1595-1601. doi:10.3201/eid2109.141665 26292017 PMC4550134

