# Supplementary material for: Mortality Associated With Influenza and Respiratory Syncytial Virus in the US, 1999-2018
Source: JAMA Netw Open. 2022 Feb 28;5(2):e220527. doi: 10.1001/jamanetworkopen.2022.0527 (PMC8886548; doi:10.1001/jamanetworkopen.2022.0527)
Supplement: Supplement. — eMethods. eTable 1. Specimens Collected and Positive Tests for Respiratory Syncytial Virus and Influenza by Season, 1999/2000 to 2017/2018, USA eTable 2. Population in Millions and Total Underlying Deaths for Pneumonia and Influenza (UPI), Respiratory (UR), and Respiratory and Circulatory (URC), and All-Causes by Age Group, 1999/2000 to 2017/2018, USA eFigure. Mortality Rates per 100,000 Population by Season, Age Group and Underlying Cause of Death eTable 3. Estimated Excess Respiratory Syncytial Virus Deaths and Mortality Rate per 100,000 Population by Season and Age Group, 1999/2000 to 2017/2018, USA eTable 4. Estimated Excess Respiratory Syncytial Virus Deaths and Mortality Rate per 100,000 Population by Season and Age Group, 2010/2011 to 2017/2018, USA When Surveillance Data Are Limited to Antigen-Based Testing Starting in 2010 eTable 5. Estimated Excess Influenza Deaths and Mortality Rate per 100,000 Population by Season and Age Group, 1999/2000 to 2017/2018, USA eTable 6. Estimated Average Annual Age-Specific Influenza Deaths by Type/Subtype, 1999/2000 to 2017/2018, USA eTable 7. Comparison With Other Published Estimates of Excess Influenza Mortality Rate for All Ages per 100,000 Population eReferences. [file jamanetwopen-e220527-s001.pdf]

Supplementary Online Content

Hansen CL, Chaves SS, Demont C, Viboud C. Mortality associated with influenza and respiratory syncytial virus in the US, 1999-2018. *JAMA Netw Open.* 2022;5(2):e220527. doi:10.1001/jamanetworkopen.2022.0527

eMethods.
eTable 1. Specimens Collected and Positive Tests for Respiratory Syncytial Virus and Influenza by Season, 1999/2000 to 2017/2018, USA
eTable 2. Population in Millions and Total Underlying Deaths for Pneumonia and Influenza (UPI), Respiratory (UR), and Respiratory and Circulatory (URC), and All-Causes by Age Group, 1999/2000 to 2017/2018, USA
eFigure. Mortality Rates per 100,000 Population by Season, Age Group and Underlying Cause of Death
eTable 3. Estimated Excess Respiratory Syncytial Virus Deaths and Mortality Rate per 100,000 Population by Season and Age Group, 1999/2000 to 2017/2018, USA
eTable 4. Estimated Excess Respiratory Syncytial Virus Deaths and Mortality Rate per 100,000 Population by Season and Age Group, 2010/2011 to 2017/2018, USA When Surveillance Data Are Limited to Antigen-Based Testing Starting in 2010
eTable 5. Estimated Excess Influenza Deaths and Mortality Rate per 100,000 Population by Season and Age Group, 1999/2000 to 2017/2018, USA
eTable 6. Estimated Average Annual Age-Specific Influenza Deaths by Type/Subtype, 1999/2000 to 2017/2018, USA
eTable 7. Comparison With Other Published Estimates of Excess Influenza Mortality Rate for All Ages per 100,000 Population
eReferences.

This supplementary material has been provided by the authors to give readers additional information about their work.

© 2022 Hansen CL et al. *JAMA Network Open.*

eMethods.

Here we provide additional details on the excess mortality regression models used to estimate the burden of influenza and RSV. We fit linear regression models to each death outcome, week, age, and location (national or by region), following:

$mr\_(t{:}c,a) = \beta_0 + ns(t) + \sum_{s=1}^{19} \beta_{1,s} * L1(flu)(t) + \beta_2 * L2(RSV)(t)$

where *mr_(t; c,a)* represents the five-week moving average of the mortality rate per 100,000 population for cause c, age group a, and week t; ns(t) is a natural cubic spline with 60 degrees of freedom representing a smooth function of time for seasonality in mortality that is not attributed to influenza or RSV. Splines have been used to model seasonally-varying excess mortality as an alternative to more conventional harmonic models as they allow for greater flexibility in baseline mortality.[1,2,3,4] We tested 2 and 3 degrees of freedom per year and used AIC to select the best model.[3,4] Flu(t) and RSV(t) are the weekly influenza and RSV circulation proxies (see main text for details); terms s1-s19 represent the severity of different influenza seasons (akin to the case fatality rate, ie the ratio of influenza cases to influenza deaths which may vary depending on the mix of strains each year). We considered different lags between viral activity and mortality, as detailed below. We also ran multiple sensitivity analyses considering different mortality outcomes and proxies of viral circulation, as described below. To account for autocorrelation in the model we used residual bootstrapping to calculate the confidence intervals. We randomly resampled the residuals from the model and added them to the predicted values using the 'car' package in R.[5] We reran the model on these new y values and repeated this process 1000 times. We used the 2.5 and 97.5 percentiles as our upper and lower confidence intervals. We chose our 5 age groups for comparison with the seminal study by Thompson et al[6] and our geographic aggregations by Health and Human Services region were based on the availability of RSV surveillance data.

Model calibration (lag between mortality and viral circulation)

We tested lags between viral circulation (both RSV and influenza) and mortality, using AIC to select the optimal lag for each age group. Prior work allowed for a lag of 0-3 weeks between viral circulation and mortality.[1,2,6,7] Because we used a 5-week moving average in our mortality data, we allowed for a lag of up to 8 weeks. We did this only for underlying respiratory mortality and used the same lag for the other causes of death. This resulted in 81 models for each age group. Below we show the lowest 10 AICs for each age group. Adjusting the lag between RSV circulation and mortality had little impact on model fit for those 5-49 years, supporting a low association between mortality and RSV circulation in this age group. There was no consensus as to the optimal lag across age groups, so we allowed each age group to have its own lag between viral circulation and mortality in the final models.

AIC Rank	<1 year		1 to 4 years		5 to 49 years		50 to 64 years		65+ years	
	Lag in weeks (influenza, RSV)	AIC	Lag in weeks (influenza, RSV)	AIC	Lag in weeks (influenza, RSV)	AIC	Lag in weeks (influenza, RSV)	AIC	Lag in weeks (influenza, RSV)	AIC
Lowest	4, 4	-4044.5	3, 3	-7763.4	3, 8	-8675.7	3, 1	-4033.0	4, 3	632.5
2nd	4, 3	-4029.2	3, 2	-7761.9	3, 7	-8675.1	3, 0	-4031.9	4, 2	638.7
3rd	4, 5	-4014.6	3, 4	-7761.6	3, 6	-8675.1	3, 2	-4026.8	4, 4	639.0
4th	5, 4	-4010.2	3, 1	-7760.5	3, 5	-8672.2	3, 3	-4019.5	4, 5	652.5
5th	3, 4	-4007.0	3, 5	-7759.5	3, 4	-8668.5	3, 4	-4014.8	4, 1	661.7
6th	3, 3	-4004.1	3, 0	-7756.8	3, 3	-8665.3	4, 3	-4012.2	4, 6	670.3
7th	5, 5	-3995.5	3, 6	-7755.8	3, 2	-8664.5	3, 5	-4011.3	4, 7	684.3
8th	4, 2	-3979.1	3, 7	-7752.9	3, 1	-8663.8	4, 2	-4009.7	4, 0	688.9
9th	4, 6	-3977.7	3, 8	-7749.8	3, 0	-8662.3	3, 6	-4008.9	4, 8	695.3
10th	5, 3	-3976.4	4, 5	-7742.6	4, 5	-8618.7	3, 7	-4007.1	5, 4	970.5

© 2022 Hansen CL et al. *JAMA Network Open.*

Influenza type/subtype analysis

We obtained weekly regional data on influenza testing and influenza like illness using the 'cdcfluview' package in R.[8] In main analyses of weekly mortality data, we use all influenza subtypes combined as a proxy for influenza incidence, but we also run a seasonal-level analysis that considers subtype-specific circulation. We applied the H1 and H3 proportions from the subtyped data to the unsubtyped influenza A samples and constructed subtype-specific influenza time series at the weekly and seasonal level. To estimate excess influenza mortality by virus subtype we regressed seasonal estimates of excess influenza mortality against the percentage positive for each subtype during that season with the intercept set to zero.[9] Excess mortality for influenza type/subtypes each season was estimated as the type/subtype coefficient multiplied by type/subtype covariate (proportion positive*ILI) for each respiratory season.

Further, to compare our results with other papers, we also ran sensitivity analyses where we model weekly mortality as a function of weekly subtype-specific incidence proxies for influenza.

Sensitivity analyses

We ran several sensitivity analyses to test model assumptions. To examine the impact of increased PCR testing for RSV over time, we ran a version of the final model which only included the proportion positive based on antigen tests from 2010 to 2018. To determine if regional level mortality counts were robust enough to present meaningful estimates, we resampled the national data using a binomial distribution to simulate expected counts in the largest (Region 4, ~20%) and smallest (Regions 8 and 10, ~4%) HHS Regions for the two extreme age groups (<1 and 65+). We fit the model to this simulated data and compared with estimates from the original models. We found that down-sampled estimates for those <1 year were not stable, but estimates in those 65+ years were robust, suggesting regional analyses in those 65+ were valid. We used the Shapiro-Wilk test to assess if our regional-level data were appropriate for ANOVA. We found that our regional data did not violate the assumption of normality but did not have constant variance. As a sensitivity analysis, we ran a non-parametric Kruskal-Wallis test. The Kruskal-Wallis test supported our conclusions that there were no statistically significant differences in influenza mortality between regions, but there were significant differences in RSV mortality. Similarly, the Kruskal-Wallis test supported our conclusion that excess UR RSV mortality was highest in Region 6 and lowest in Region 10.

Missing data

Where death certificates were missing the exact date of death (0.003%), the fifteenth of the month was used. Death certificates where age of deceased was missing were excluded from analysis (0.01%). Influenza surveillance for Region 10 was missing prior to September 21, 2008. We used neighboring Region 8 which had the highest correlation after 2008 to fill in the missing weeks.

Comparing influenza estimates with other studies

To compare our influenza estimates with other studies we tested the impact of adjustments to our model on the estimated average annual mortality rates. To simplify these comparisons, we aggregated all age groups and converted everything to rates per 100,000 population. When other studies provided estimates by year, we compared averages only for overlapping years. For comparing to studies estimating the impact of the 2009 pandemic, we calculated the rate for the same months covered in those analyses.

© 2022 Hansen CL et al. *JAMA Network Open.*

Our comparison analyses include the following models:

(a) Our final model as described in the main text

$mr\_(t{:}c,a) = \beta_0 + ns(t) + \sum_{s=1}^{19} \beta_{1,s}*L1(flu)(t) + \beta_2*L2(RSV)(t)$

(b) A model similar to the above but with all ages aggregated rather than summing results from age-specific models

$mr\_(t{:}c) = \beta_0 + ns(t) + \sum_{s=1}^{19} \beta_{1,s}*L1(flu)(t) + \beta_2*L2(RSV)(t)$

(c) A model with sine/cosine terms instead of a spline term to model baseline seasonality

$mr\_(t{:}c,a) = \beta_0 + ns(t) + \sum_{s=1}^{19} \beta_{1,s}*L1(flu)(t) + \beta_2*L2(RSV)(t) + \beta_3*(t) + \beta_4*(t^2) + \beta_5*sin(2\pi/52*t) + \beta_6*cos(2\pi/52*t)$

(d) A model which does not include a term for RSV

$mr\_(t{:}c,a) = \beta_0 + ns(t) + \sum_{s=1}^{19} \beta_{1,s}*L1(flu)(t)$

(e) A model which has individual terms for each influenza type/subtype

$mr\_(t{:}c,a) = \beta_0 + ns(t) + \beta_1* L1(A/H1)(t) + \beta_2* L1(A/H3)(t) + \beta_3* L1(A/H1N1pdm09)(t) + \beta_4* L1(B)(t) + \beta_5*L2(RSV)(t)$

(f) A model which includes contributing causes of death

$mr\_(t{:}c[contributing],a) = \beta_0 + ns(t) + \sum_{s=1}^{19} \beta_{1,s}*L1(flu)(t) + \beta_2*L2(RSV)(t)$

We used the same causes of death described in each paper. For estimates produced with the multiplier method we compared with all-cause mortality unless the paper specifically indicated that more specific causes of hospitalization and death were considered. All models use underlying cause of death except model (f) which includes contributing causes of death. These results are described in eTable 7 and summarized in the discussion of the main text.

© 2022 Hansen CL et al. *JAMA Network Open.*

eTable 1. Specimens Collected and Positive Tests for Respiratory Syncytial Virus and Influenza by Season, 1999/2000 to 2017/2018, USA

Season	RSV Specimens Tested	Positive Tests (%)	Antigen Tests (%)	Positive Antigen Tests (%)	Culture Tests (%)	Positive Culture Tests (%)	PCR Tests (%)	Positive PCR Tests (%)	Influenza Specimens Tested	Positive Influenza Tests (%)	A (H1) Positive Tests (%)	A (H3) Positive Tests (%)	H1N1pdm09 Positive Tests (%)	Influenza B Positive Tests (%)
1999/2000	127923	19063 (14.9)							110622	14695 (13.3)	559 (0.5)	14033 (12.7)	0(0.0)	103 (0.1)
2000/2001	142261	18681 (13.1)							102363	10511 (10.3)	5438 (5.3)	164 (0.2)	0(0.0)	4909 (4.8)
2001/2002	127117	18611 (14.6)							114910	16541 (14.4)	252 (0.2)	13926 (12.1)	0(0.0)	2363 (2.1)
2002/2003	124760	14287 (11.5)							95754	9822 (10.3)	4195 (4.4)	1536 (1.6)	0(0.0)	4091 (4.3)
2003/2004	172252	21154 (12.3)							150645	25089 (16.7)	7 (0.0)	24793 (16.5)	0(0.0)	289 (0.2)
2004/2005	209185	22641 (10.8)							182183	24568 (13.5)	49 (0.0)	18320 (10.1)	0(0.0)	6199 (3.4)
2005/2006	232132	27067 (11.7)							181790	21230 (11.7)	1521 (0.8)	15311 (8.4)	0(0.0)	4398 (2.4)
2006/2007	384147	54189 (14.1)							204107	24964 (12.2)	13512 (6.6)	6124 (3.0)	0(0.0)	5328 (2.6)
2007/2008	603196	85120 (14.1)							258533	42390 (16.4)	7203 (2.8)	22973 (8.9)	0(0.0)	12214 (4.7)
2008/2009	694521	86580 (12.5)							398419	74579 (18.7)	12410 (3.1)	6969 (1.7)	43939 (11.0)	11264 (2.8)
2009/2010	746132[a]	82432 (11.0)	226320 (65.6)	45953 (20.3)	61537 (17.8)	3293 (5.4)	57261 (16.6)	7217(12.6)	536407	110176 (20.5)	151 (0.0)	318 (0.1)	109262 (20.4)	441 (0.1)
2010/2011	656704	83605 (12.7)	395508 (60.2)	66743 (16.9)	108725 (16.6)	3564 (3.3)	152471 (23.2)	13298(8.7)	299325	56143 (18.8)	1 (0.0)	26080 (8.7)	15419 (5.2)	14642 (4.9)
2011/2012	569664	69013 (12.1)	334239 (58.7)	53457 (16.0)	89001 (15.6)	2735 (3.1)	146424 (25.7)	12821(8.8)	224251	25689 (11.5)	0 (0.0)	15057 (6.7)	6132 (2.7)	4500 (2.0)
2012/2013	727409	92797 (12.8)	353662 (48.6)	60308 (17.1)	90288 (12.4)	2950 (3.3)	283459 (39.0)	29539(10.4)	391797	80132 (20.5)	0 (0.0)	53132 (13.6)	3048 (0.8)	23952 (6.1)
2013/2014	465356	46831 (10.1)	180475 (38.8)	25553 (14.2)	51013 (11.0)	1289 (2.5)	233868 (50.3)	19989(8.5)	382100	58542 (15.3)	0 (0.0)	5389 (1.4)	44805 (11.7)	8348 (2.2)
2014/2015	652785	64396 (9.9)	183919 (28.2)	27731 (15.1)	46315 (7.1)	1329 (2.9)	422551 (64.7)	35336(8.4)	791051	129080 (16.3)	0 (0.0)	106516 (13.5)	551 (0.1)	22013 (2.8)
2015/2016	765860	60931 (8.0)	163387 (21.3)	22636 (13.9)	36149 (4.7)	578 (1.6)	566324 (73.9)	37717(6.7)	875986	99380 (11.3)	0 (0.0)	14002 (1.6)	53498 (6.1)	31880 (3.6)
2016/2017	890214	74814 (8.4)	152164 (17.1)	20931 (13.8)	27719 (3.1)	344 (1.2)	710331 (79.8)	53539(7.5)	1156066	176690 (15.3)	0 (0.0)	122658 (10.6)	4000 (0.3)	50032 (4.3)
2017/2018	1035672	88236 (8.5)	142148 (13.7)	21874 (15.4)	28104 (2.7)	434 (1.5)	865420 (83.6)	65928(7.6)	1542596	295346 (19.1)	0 (0.0)	167832 (10.9)	32568 (2.1)	94946 (6.2)

[a] Information on test type only available for 345,118 samples during this season

© 2022 Hansen CL et al. *JAMA Network Open.*

eTable 2. Population in Millions and Total Underlying Deaths for Pneumonia and Influenza (UPI), Respiratory (UR), and Respiratory and Circulatory (URC), and All-Causes by Age Group, 1999/2000 to 2017/2018, USA

Age in years		1999/00	2000/01	2001/02	2002/203	2003/04	2004/05	2005/06	2006/07	2007/08	2008/09	2009/10	2010/11	2011/12	2012/13	2013/14	2014/15	2015/16	2016/17	2017/18
<1	Pop[a]	3.771	3.831	3.986	3.925	3.949	3.986	3.977	4.014	4.119	4.103	3.974	3.922	3.932	3.897	3.901	3.925	3.955	3.925	3.865
	All[b]	27879	27481	27637	27479	28143	28172	27982	28668	28516	27547	25017	23954	23544	23321	23095	23664	22914	22524	21593
	URC[c]	1295	1285	1313	1233	1272	1239	1173	1259	1207	1146	1157	1069	964	1021	962	966	963	938	831
	UR[d]	667	649	646	634	688	667	653	669	595	573	608	573	482	536	505	537	533	476	405
	UPI[e]	293	289	272	265	298	292	247	235	222	238	226	199	149	186	188	180	169	154	156
1 to 4	Pop[a]	15.237	15.222	15.187	15.378	15.516	15.67	15.809	15.791	15.871	16.029	16.13	16.124	16.045	15.933	15.801	15.8	15.816	15.85	15.882
	All[b]	4991	5079	4903	4870	4965	4660	4661	4593	4698	4642	4236	4361	4118	4082	3920	3927	3920	3872	3850
	URC[c]	490	543	539	520	584	504	536	477	585	507	532	523	464	479	497	470	471	449	461
	UR[d]	260	289	269	291	358	266	313	259	328	284	327	300	248	277	301	275	279	252	294
	UPI[e]	106	116	110	105	175	106	120	102	136	111	134	115	79	110	98	105	97	95	133
5 to 49	Pop[a]	183.155	184.463	185.344	185.985	186.303	186.697	186.976	187.518	187.763	187.999	188.24	188.254	188.182	188.301	188.407	188.499	188.792	189.35	189.863
	All[b]	235710	239242	246625	245888	250963	242531	244748	241263	235566	232414	222956	219188	214437	214967	213775	222899	227997	240675	239911
	URC[c]	47085	47651	47887	49013	50020	48219	47871	46593	46235	45281	46158	43854	41377	41458	42224	41535	40883	41326	41591
	UR[d]	7139	7127	7114	7159	7351	7192	6908	6814	7121	7005	8750	7225	6249	6741	7893	6664	6850	6573	7150
	UPI[e]	2489	2375	2435	2390	2590	2465	2304	2179	2467	2311	3847	2542	1923	2284	3217	2238	2369	2180	2625
50 to 64	Pop[a]	40.437	41.933	43.502	45.137	46.784	48.529	50.364	52.13	53.884	55.4	57.003	58.717	60.207	60.72	61.398	62.069	62.575	62.77	62.795
	All[b]	321983	332064	340978	351206	366895	369376	379351	387763	397302	409931	414134	427103	428964	444090	450998	468829	465287	471229	473408
	URC[c]	122537	123964	125019	127745	131366	131075	131750	133210	135802	138272	140324	142735	141287	148838	152304	157394	157009	159955	163092
	UR[d]	20440	20886	21096	21817	23672	24099	23559	24530	26321	27176	28842	29268	28297	31559	33190	33819	34080	34551	36857
	UPI[e]	3786	3596	3918	3974	4432	4588	4284	4297	4819	4700	5878	5361	4832	5788	7208	6044	6339	5871	7506
65+	Pop[a]	34.605	34.872	35.089	35.318	35.655	35.991	36.432	36.942	37.599	38.543	39.38	40.226	41.089	42.856	44.342	45.857	47.339	48.878	50.417
	All[b]	1766111	1797476	1791821	1774316	1817009	1768879	1743038	1745723	1776090	1775709	1758061	1818253	1802820	1900731	1868700	2006915	1954100	2026983	2077036
	URC[c]	985349	989724	973293	947879	959644	916729	873209	857604	861840	845839	824198	842363	820827	868760	844167	915011	882310	915826	938866
	UR[d]	197208	200064	202632	195428	209307	204638	193176	192454	205037	200365	198446	209371	202458	222582	209385	233820	217492	231806	238994
	UPI[e]	57352	55377	58247	53798	59331	55008	49775	46778	48454	43549	42491	45325	41840	48879	42979	50544	41830	45500	50560
Total	Pop[a]	277.205	280.321	283.108	285.743	288.207	290.873	293.558	296.395	299.236	302.074	304.727	307.243	309.455	311.707	313.849	316.15	318.477	320.773	322.822
	All[b]	2356674	2401342	2411964	2403759	2467975	2413618	2399780	2408010	2442172	2450243	2424404	2492859	2473883	2587191	2560488	2726234	2674218	2765283	2815798
	URC[c]	1156756	1163167	1148051	1126390	1142886	1097766	1054539	1039143	1045669	1031045	1012369	1030544	1004919	1060556	1040154	1115376	1081636	1118494	1144841
	UR[d]	225714	229015	231757	225329	241376	236862	224609	224726	239402	235403	236973	246737	237734	261695	251274	275115	259234	273658	283700
	UPI[e]	64026	61753	64982	60532	66826	62459	56730	53591	56098	50909	52576	53542	48823	57247	53690	59111	50804	53800	60980

[a] Population in millions
[b] All-cause deaths
[c] Underlying respiratory and circulatory deaths
[d] Underlying respiratory deaths
[e] Underlying pneumonia and influenza deaths

© 2022 Hansen CL et al. *JAMA Network Open.*

eFigure. Mortality Rates per 100,000 Population by Season, Age Group and Underlying Cause of Death

Black points represent the annual mortality rate per 100,000 population for underlying pneumonia and influenza (UPI), underlying respiratory (UR), underling respiratory and circulatory (URC), and all-causes for each season from 1999/2000 to 2017/2018. The solid grey line represents a loess fit. The dashed blue line represents a linear fit. The p-value in the bottom left-hand corner corresponds to the linear fit.

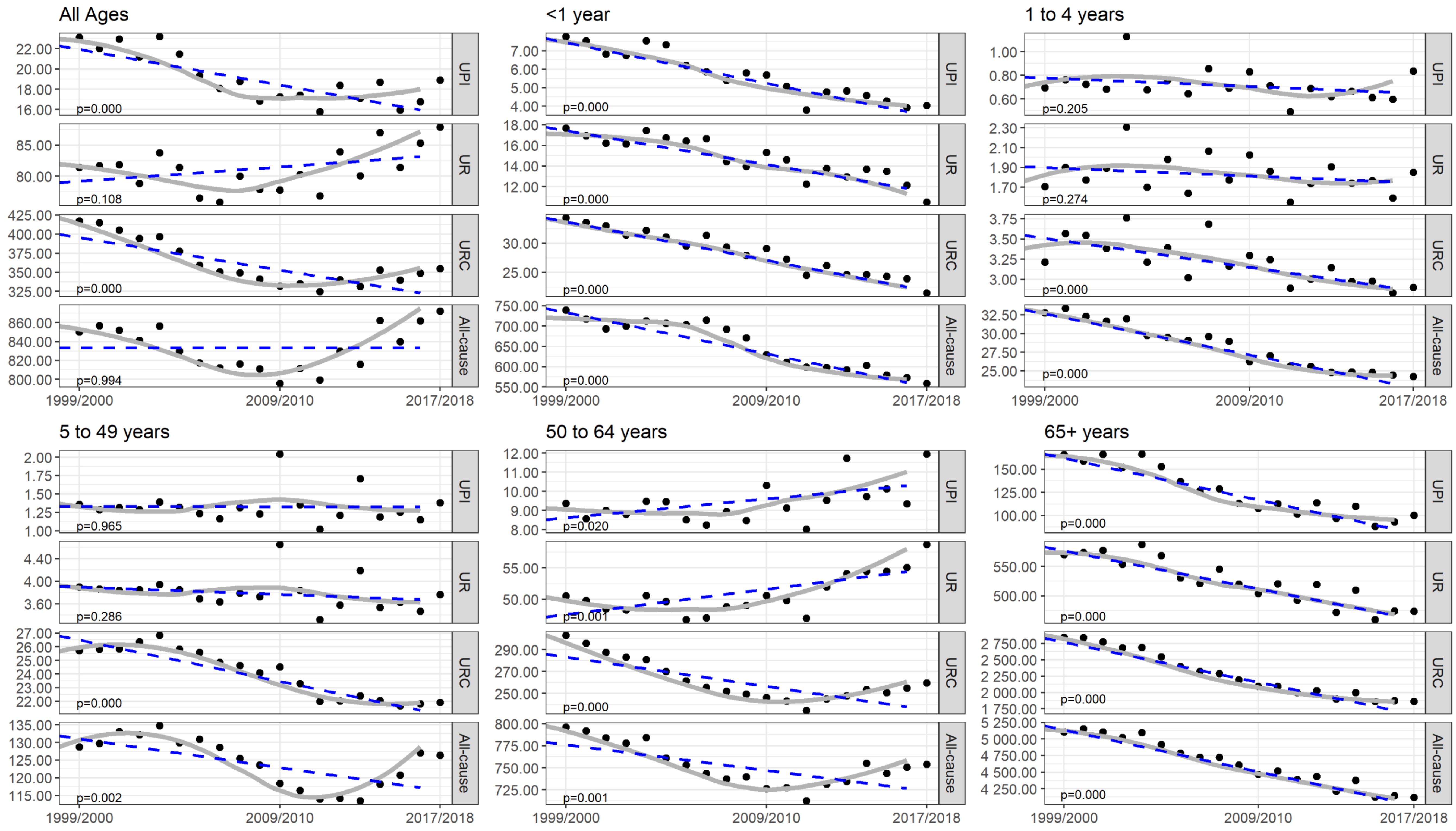

© 2022 Hansen CL et al. *JAMA Network Open.*

eTable 3. Estimated Excess Respiratory Syncytial Virus Deaths and Mortality Rate per 100,000 Population by Season and Age Group, 1999/2000 to 2017/2018, USA

Cause of Death	Season	Deaths in <1 year (95% CI)	Deaths in 1 to 4 years (95% CI)	Deaths in 5 to 49 years (95%CI)	Deaths in 50 to 64 years (95% CI)	Deaths in 65+ years (95% CI)	Total RSV deaths (95% CI)	RSV mortality rate per 100,000 population (95% CI)	RSV mortality rate per 100,000 population, age-standardized to 2017/2018 population (95% CI)
UPI	1999/2000	56 (44-66)	6 (-2-13)	74 (5-145)	234 (148-322)	2892 (2186-3598)	3262 (2382-4143)	1.2 (0.9-1.5)	1.5 (1.1-1.9)
UPI	2000/2001	49 (39-58)	5 (-2-11)	62 (4-121)	212 (135-292)	2521 (1906-3136)	2849 (2082-3618)	1.0 (0.7-1.3)	1.3 (0.9-1.6)
UPI	2001/2002	53 (42-62)	5 (-2-12)	65 (4-127)	229 (145-315)	2636 (1993-3279)	2988 (2182-3795)	1.1 (0.8-1.3)	1.3 (1.0-1.7)
UPI	2002/2003	48 (38-56)	5 (-2-11)	60 (4-116)	216 (137-298)	2427 (1834-3018)	2755 (2012-3499)	1.0 (0.7-1.2)	1.2 (0.9-1.5)
UPI	2003/2004	51 (40-60)	5 (-2-11)	65 (4-127)	238 (151-328)	2599 (1965-3233)	2958 (2159-3759)	1.0 (0.7-1.3)	1.3 (0.9-1.6)
UPI	2004/2005	40 (32-47)	4 (-1-9)	49 (3-96)	192 (122-265)	2034 (1537-2529)	2319 (1693-2945)	0.8 (0.6-1.0)	1.0 (0.7-1.3)
UPI	2005/2006	43 (35-51)	4 (-2-10)	55 (4-106)	218 (138-300)	2261 (1709-2813)	2582 (1884-3280)	0.9 (0.6-1.1)	1.1 (0.8-1.4)
UPI	2006/2007	53 (42-63)	5 (-2-12)	66 (5-128)	276 (175-380)	2788 (2108-3468)	3188 (2328-4051)	1.1 (0.8-1.4)	1.3 (1.0-1.7)
UPI	2007/2008	56 (44-66)	5 (-2-12)	68 (5-133)	290 (184-399)	2897 (2190-3603)	3316 (2421-4213)	1.1 (0.8-1.4)	1.3 (1.0-1.7)
UPI	2008/2009	54 (43-63)	5 (-2-12)	66 (5-129)	289 (183-398)	2876 (2174-3577)	3290 (2403-4179)	1.1 (0.8-1.4)	1.3 (1.0-1.7)
UPI	2009/2010	50 (40-58)	5 (-2-12)	63 (4-122)	283 (180-390)	2798 (2115-3480)	3198 (2336-4062)	1.0 (0.8-1.3)	1.2 (0.9-1.6)
UPI	2010/2011	48 (38-56)	5 (-2-11)	61 (4-118)	286 (181-394)	2800 (2116-3483)	3199 (2338-4062)	1.0 (0.8-1.3)	1.2 (0.9-1.5)
UPI	2011/2012	49 (39-58)	5 (-2-12)	62 (4-121)	300 (190-413)	2919 (2207-3631)	3335 (2439-4234)	1.1 (0.8-1.4)	1.2 (0.9-1.6)
UPI	2012/2013	50 (39-58)	5 (-2-12)	65 (4-126)	306 (194-422)	3096 (2340-3851)	3521 (2577-4468)	1.1 (0.8-1.4)	1.3 (0.9-1.6)
UPI	2013/2014	43 (34-50)	4 (-2-10)	55 (4-106)	264 (168-364)	2736 (2068-3404)	3102 (2272-3933)	1.0 (0.7-1.3)	1.1 (0.8-1.4)
UPI	2014/2015	41 (32-48)	4 (-1-9)	53 (4-103)	255 (162-351)	2702 (2042-3360)	3054 (2239-3872)	1.0 (0.7-1.2)	1.0 (0.8-1.3)
UPI	2015/2016	35 (28-41)	4 (-1-8)	45 (3-87)	220 (140-303)	2387 (1804-2969)	2690 (1974-3408)	0.8 (0.6-1.1)	0.9 (0.6-1.1)
UPI	2016/2017	35 (28-42)	4 (-1-8)	46 (3-89)	224 (142-309)	2496 (1887-3104)	2805 (2059-3552)	0.9 (0.6-1.1)	0.9 (0.7-1.1)
UPI	2017/2018	35 (28-41)	4 (-1-8)	45 (3-88)	225 (143-310)	2579 (1949-3207)	2887 (2121-3654)	0.9 (0.7-1.1)	0.9 (0.7-1.1)
UR	1999/2000	115 (97-134)	24 (13-36)	156 (71-251)	475 (289-675)	6319 (4747-7951)	7089 (5217-9047)	2.6 (1.9-3.3)	3.2 (2.3-4.0)
UR	2000/2001	100 (85-117)	21 (11-31)	131 (60-210)	431 (262-612)	5508 (4137-6930)	6191 (4555-7901)	2.2 (1.6-2.8)	2.7 (2.0-3.5)
UR	2001/2002	108 (92-126)	22 (11-32)	137 (63-220)	464 (282-659)	5760 (4327-7247)	6491 (4775-8285)	2.3 (1.7-2.9)	2.9 (2.1-3.6)
UR	2002/2003	97 (83-114)	20 (11-30)	125 (57-201)	440 (267-625)	5302 (3982-6671)	5984 (4400-7640)	2.1 (1.5-2.7)	2.6 (1.9-3.3)
UR	2003/2004	104 (89-122)	22 (11-32)	137 (63-220)	483 (294-687)	5678 (4265-7144)	6424 (4721-8205)	2.2 (1.6-2.8)	2.8 (2.0-3.5)
UR	2004/2005	81 (69-95)	17 (9-25)	103 (47-166)	390 (237-554)	4443 (3337-5590)	5035 (3700-6431)	1.7 (1.3-2.2)	2.1 (1.6-2.7)
UR	2005/2006	89 (76-104)	19 (10-28)	114 (52-184)	443 (269-629)	4940 (3711-6216)	5605 (4118-7161)	1.9 (1.4-2.4)	2.4 (1.7-3.0)
UR	2006/2007	109 (93-128)	23 (12-34)	138 (63-222)	560 (340-796)	6092 (4576-7665)	6922 (5084-8844)	2.3 (1.7-3.0)	2.9 (2.1-3.7)
UR	2007/2008	115 (98-134)	23 (12-34)	143 (66-230)	588 (358-836)	6328 (4753-7962)	7198 (5287-9197)	2.4 (1.8-3.1)	2.9 (2.2-3.7)
UR	2008/2009	111 (94-130)	23 (12-34)	139 (64-223)	586 (357-834)	6283 (4719-7905)	7142 (5246-9126)	2.4 (1.7-3.0)	2.8 (2.1-3.6)
UR	2009/2010	102 (87-119)	22 (11-32)	132 (60-211)	575 (349-817)	6112 (4591-7691)	6942 (5099-8870)	2.3 (1.7-2.9)	2.7 (2.0-3.4)
UR	2010/2011	99 (84-115)	21 (11-32)	128 (59-205)	581 (353-825)	6117 (4594-7696)	6945 (5101-8873)	2.3 (1.7-2.9)	2.6 (1.9-3.4)
UR	2011/2012	101 (86-118)	22 (11-32)	130 (60-210)	608 (370-865)	6378 (4791-8025)	7240 (5318-9250)	2.3 (1.7-3.0)	2.7 (2.0-3.4)
UR	2012/2013	102 (87-119)	22 (11-32)	136 (62-218)	622 (378-884)	6763 (5080-8510)	7645 (5619-9763)	2.5 (1.8-3.1)	2.7 (2.0-3.5)
UR	2013/2014	87 (74-102)	19 (10-27)	115 (53-184)	536 (326-762)	5978 (4490-7522)	6734 (4953-8597)	2.1 (1.6-2.7)	2.3 (1.7-3.0)
UR	2014/2015	84 (71-98)	18 (9-26)	111 (51-178)	518 (315-736)	5902 (4433-7426)	6632 (4880-8465)	2.1 (1.5-2.7)	2.2 (1.6-2.9)
UR	2015/2016	72 (61-84)	15 (8-22)	93 (43-150)	447 (272-636)	5215 (3917-6562)	5843 (4301-7454)	1.8 (1.4-2.3)	1.9 (1.4-2.4)
UR	2016/2017	72 (62-85)	15 (8-23)	96 (44-154)	455 (277-647)	5453 (4096-6860)	6092 (4486-7769)	1.9 (1.4-2.4)	1.9 (1.4-2.5)
UR	2017/2018	71 (61-83)	16 (8-23)	95 (43-152)	456 (278-649)	5634 (4232-7088)	6272 (4621-7996)	1.9 (1.4-2.5)	1.9 (1.4-2.5)

© 2022 Hansen CL et al. *JAMA Network Open.*

eTable 3. Estimated Excess Respiratory Syncytial Virus Deaths and Mortality Rate per 100,000 Population by Season and Age Group, 1999/2000 to 2017/2018, USA, continued

Cause of Death	Season	Deaths in <1 year (95% CI)	Deaths in 1 to 4 years (95% CI)	Deaths in 5 to 49 years (95%CI)	Deaths in 50 to 64 years (95% CI)	Deaths in 65+ years (95% CI)	Total RSV deaths (95% CI)	RSV mortality rate per 100,000 population (95% CI)	RSV mortality rate per 100,000 population, age-standardized to 2017/2018 population (95% CI)
URC	1999/2000	138 (112-159)	17 (2-32)	-312 (-509--106)	3614 (3130-4091)	13732 (10192-17714)	17188 (12928-21890)	6.2 (4.7-7.9)	7.9 (6.0-10.0)
URC	2000/2001	121 (98-140)	15 (1-27)	-262 (-427--89)	3278 (2840-3711)	11968 (8884-15440)	15120 (11396-19229)	5.4 (4.1-6.9)	6.8 (5.2-8.7)
URC	2001/2002	130 (106-150)	15 (2-28)	-274 (-447--93)	3530 (3058-3996)	12517 (9290-16147)	15917 (12009-20228)	5.6 (4.2-7.1)	7.1 (5.4-9.0)
URC	2002/2003	117 (95-135)	14 (1-26)	-251 (-409--85)	3345 (2897-3786)	11520 (8551-14862)	14745 (11136-18724)	5.2 (3.9-6.6)	6.5 (4.9-8.2)
URC	2003/2004	125 (102-145)	15 (2-28)	-274 (-446--93)	3675 (3183-4160)	12339 (9158-15917)	15880 (11999-20157)	5.5 (4.2-7.0)	6.9 (5.2-8.7)
URC	2004/2005	98 (80-113)	12 (1-22)	-207 (-337--70)	2968 (2571-3360)	9654 (7166-12454)	12525 (9481-15879)	4.3 (3.3-5.5)	5.3 (4.1-6.8)
URC	2005/2006	107 (87-124)	13 (1-24)	-229 (-374--78)	3369 (2919-3814)	10735 (7968-13848)	13995 (10601-17732)	4.8 (3.6-6.0)	5.9 (4.5-7.4)
URC	2006/2007	131 (107-152)	16 (2-30)	-276 (-451--94)	4259 (3689-4822)	13237 (9825-17076)	17367 (13172-21985)	5.9 (4.4-7.4)	7.1 (5.4-9.0)
URC	2007/2008	138 (112-159)	16 (2-30)	-286 (-467--98)	4478 (3879-5069)	13751 (10207-17739)	18096 (13732-22900)	6.0 (4.6-7.7)	7.3 (5.5-9.2)
URC	2008/2009	133 (109-154)	16 (2-30)	-278 (-454--95)	4462 (3866-5052)	13653 (10134-17612)	17986 (13656-22753)	6.0 (4.5-7.5)	7.1 (5.4-8.9)
URC	2009/2010	122 (100-142)	15 (2-28)	-263 (-429--90)	4373 (3788-4951)	13282 (9858-17134)	17529 (13318-22165)	5.8 (4.4-7.3)	6.7 (5.1-8.5)
URC	2010/2011	118 (97-137)	15 (2-28)	-255 (-417--87)	4417 (3827-5001)	13292 (9866-17146)	17587 (13374-22225)	5.7 (4.4-7.2)	6.6 (5.0-8.3)
URC	2011/2012	121 (99-140)	15 (2-28)	-261 (-426--89)	4629 (4010-5240)	13860 (10287-17880)	18364 (13972-23199)	5.9 (4.5-7.5)	6.7 (5.1-8.5)
URC	2012/2013	122 (100-142)	15 (2-28)	-272 (-443--93)	4732 (4099-5357)	14697 (10908-18959)	19294 (14666-24394)	6.2 (4.7-7.8)	6.8 (5.2-8.6)
URC	2013/2014	105 (85-121)	13 (1-24)	-230 (-374--78)	4078 (3533-4617)	12990 (9642-16757)	16956 (12887-21442)	5.4 (4.1-6.8)	5.8 (4.4-7.4)
URC	2014/2015	100 (82-116)	12 (1-23)	-221 (-361--76)	3941 (3414-4462)	12826 (9520-16545)	16658 (12656-21071)	5.3 (4.0-6.7)	5.6 (4.2-7.1)
URC	2015/2016	86 (71-100)	11 (1-20)	-187 (-305--64)	3403 (2948-3853)	11332 (8411-14619)	14645 (11126-18528)	4.6 (3.5-5.8)	4.8 (3.6-6.0)
URC	2016/2017	87 (71-101)	11 (1-20)	-191 (-312--65)	3466 (3002-3924)	11848 (8794-15284)	15220 (11556-19263)	4.7 (3.6-6.0)	4.8 (3.7-6.1)
URC	2017/2018	86 (70-99)	11 (1-20)	-190 (-310--65)	3474 (3009-3933)	12242 (9086-15792)	15622 (11857-19779)	4.8 (3.7-6.1)	4.8 (3.7-6.1)
All Cause	1999/2000	127 (-5-251)	203 (144-258)	-2220 (-2986--1509)	5911 (5144-6668)	20175 (14753-25888)	24196 (17051-31556)	8.7 (6.2-11.4)	11.3 (8.2-14.6)
All Cause	2000/2001	111 (-4-220)	175 (124-223)	-1862 (-2505--1266)	5362 (4666-6049)	17584 (12859-22564)	21371 (15141-27790)	7.6 (5.4-9.9)	9.9 (7.2-12.6)
All Cause	2001/2002	120 (-4-236)	182 (129-231)	-1949 (-2622--1325)	5774 (5025-6514)	18389 (13448-23597)	22516 (15975-29253)	8.0 (5.6-10.3)	10.2 (7.4-13.1)
All Cause	2002/2003	108 (-4-213)	168 (119-214)	-1782 (-2397--1212)	5471 (4761-6172)	16926 (12378-21719)	20891 (14857-27106)	7.3 (5.2-9.5)	9.4 (6.8-12.0)
All Cause	2003/2004	116 (-4-228)	180 (128-229)	-1947 (-2619--1324)	6011 (5231-6781)	18128 (13257-23261)	22488 (15992-29176)	7.8 (5.5-10.1)	9.9 (7.2-12.7)
All Cause	2004/2005	90 (-3-178)	141 (100-179)	-1471 (-1978--1000)	4855 (4225-5477)	14184 (10372-18200)	17800 (12716-23035)	6.1 (4.4-7.9)	7.7 (5.6-9.9)
All Cause	2005/2006	99 (-4-195)	156 (111-199)	-1629 (-2191--1108)	5511 (4796-6217)	15771 (11533-20237)	19909 (14245-25741)	6.8 (4.9-8.8)	8.5 (6.1-10.8)
All Cause	2006/2007	121 (-4-240)	190 (135-241)	-1966 (-2645--1337)	6966 (6062-7859)	19448 (14222-24955)	24759 (17769-31958)	8.4 (6.0-10.8)	10.3 (7.5-13.2)
All Cause	2007/2008	127 (-5-251)	195 (138-248)	-2038 (-2741--1386)	7324 (6374-8262)	20203 (14774-25925)	25812 (18540-33300)	8.6 (6.2-11.1)	10.5 (7.6-13.5)
All Cause	2008/2009	123 (-4-243)	191 (135-242)	-1978 (-2661--1345)	7299 (6352-8235)	20059 (14668-25739)	25694 (18490-33113)	8.5 (6.1-11.0)	10.2 (7.4-13.0)
All Cause	2009/2010	113 (-4-223)	183 (129-232)	-1873 (-2519--1273)	7153 (6225-8069)	19514 (14270-25040)	25090 (18101-32291)	8.2 (5.9-10.6)	9.7 (7.0-12.4)
All Cause	2010/2011	109 (-4-216)	179 (127-227)	-1816 (-2443--1235)	7226 (6288-8152)	19528 (14280-25058)	25225 (18248-32417)	8.2 (5.9-10.6)	9.5 (6.9-12.2)
All Cause	2011/2012	112 (-4-221)	182 (129-231)	-1856 (-2497--1262)	7571 (6589-8541)	20363 (14891-26130)	26372 (19107-33860)	8.5 (6.2-10.9)	9.7 (7.0-12.4)
All Cause	2012/2013	113 (-4-223)	183 (130-233)	-1932 (-2599--1314)	7740 (6736-8732)	21592 (15790-27707)	27697 (20053-35581)	8.9 (6.4-11.4)	9.8 (7.1-12.6)
All Cause	2013/2014	97 (-4-191)	155 (110-198)	-1633 (-2196--1110)	6671 (5806-7526)	19085 (13956-24489)	24375 (17672-31293)	7.8 (5.6-10.0)	8.4 (6.1-10.8)
All Cause	2014/2015	93 (-3-183)	148 (105-189)	-1575 (-2119--1071)	6447 (5610-7273)	18843 (13780-24180)	23956 (17373-30753)	7.6 (5.5-9.7)	8.0 (5.8-10.3)
All Cause	2015/2016	80 (-3-158)	127 (90-162)	-1329 (-1787--904)	5566 (4844-6280)	16649 (12175-21364)	21094 (15319-27059)	6.6 (4.8-8.5)	6.9 (5.0-8.8)
All Cause	2016/2017	80 (-3-159)	129 (91-164)	-1361 (-1831--926)	5669 (4933-6395)	17407 (12730-22337)	21924 (15920-28129)	6.8 (5.0-8.8)	7.0 (5.1-8.9)
All Cause	2017/2018	79 (-3-156)	129 (92-165)	-1350 (-1816--918)	5682 (4945-6410)	17985 (13152-23078)	22526 (16369-28891)	7.0 (5.1-8.9)	7.0 (5.1-8.9)

© 2022 Hansen CL et al. *JAMA Network Open.*

eTable 4. Estimated Excess Respiratory Syncytial Virus Deaths and Mortality Rate per 100,000 Population by Season and Age Group, 2010/2011 to 2017/2018, USA When Surveillance Data Are Limited to Antigen-Based Testing Starting in 2010

Cause of Death	Season	Deaths in <1 year (95% CI)	Deaths in 1 to 4 years (95% CI)	Deaths in 5 to 49 years (95%CI)	Deaths in 50 to 64 years (95% CI)	Deaths in 65+ years (95% CI)	Total RSV deaths (95% CI)	RSV mortality rate per 100,000 population (95% CI)	RSV mortality rate per 100,000 population, age-standardized to 2017/2018 population (95% CI)
UPI	2010/2011	52 (42-63)	5 (-3-12)	90 (31-152)	333 (198-454)	3359 (2544-4195)	3840 (2813-4877)	1.2 (0.9-1.6)	1.5 (1.1-1.8)
UPI	2011/2012	54 (43-64)	5 (-3-12)	92 (32-155)	350 (208-477)	3499 (2650-4370)	3999 (2930-5078)	1.3 (0.9-1.6)	1.5 (1.1-1.9)
UPI	2012/2013	55 (44-66)	5 (-3-12)	98 (34-165)	365 (217-498)	3785 (2866-4726)	4308 (3159-5468)	1.4 (1.0-1.8)	1.5 (1.1-2.0)
UPI	2013/2014	48 (39-58)	4 (-3-11)	84 (29-142)	322 (192-439)	3426 (2595-4279)	3885 (2851-4928)	1.2 (0.9-1.6)	1.4 (1.0-1.7)
UPI	2014/2015	50 (40-60)	4 (-3-11)	88 (30-149)	335 (200-457)	3650 (2764-4557)	4127 (3031-5235)	1.3 (1.0-1.7)	1.4 (1.0-1.8)
UPI	2015/2016	50 (40-60)	4 (-3-11)	86 (30-145)	335 (200-458)	3736 (2830-4666)	4212 (3096-5340)	1.3 (1.0-1.7)	1.4 (1.0-1.7)
UPI	2016/2017	49 (39-59)	4 (-3-11)	86 (30-146)	335 (200-458)	3838 (2906-4792)	4313 (3173-5466)	1.3 (1.0-1.7)	1.4 (1.0-1.7)
UPI	2017/2018	55 (44-66)	5 (-3-12)	97 (33-164)	382 (228-521)	4498 (3406-5617)	5037 (3708-6380)	1.6 (1.1-2.0)	1.6 (1.1-2.0)
UR	2010/2011	109 (93-125)	21 (10-34)	176 (94-257)	716 (451-990)	7487 (5851-9111)	8510 (6499-10517)	2.8 (2.1-3.4)	3.2 (2.5-4.0)
UR	2011/2012	111 (95-128)	22 (10-35)	178 (96-261)	752 (474-1039)	7799 (6095-9491)	8862 (6769-10953)	2.9 (2.2-3.5)	3.3 (2.5-4.1)
UR	2012/2013	115 (98-132)	22 (10-36)	190 (102-278)	785 (494-1085)	8435 (6592-10265)	9547 (7296-11795)	3.1 (2.3-3.8)	3.4 (2.6-4.2)
UR	2013/2014	100 (86-115)	19 (9-31)	164 (88-239)	692 (436-957)	7636 (5967-9292)	8612 (6586-10634)	2.7 (2.1-3.4)	3.0 (2.3-3.7)
UR	2014/2015	104 (89-120)	20 (9-32)	171 (92-250)	721 (454-996)	8134 (6356-9898)	9150 (7000-11296)	2.9 (2.2-3.6)	3.1 (2.4-3.8)
UR	2015/2016	104 (89-119)	20 (9-32)	167 (90-245)	721 (454-997)	8327 (6507-10133)	9340 (7149-11526)	2.9 (2.2-3.6)	3.1 (2.3-3.8)
UR	2016/2017	102 (88-118)	20 (9-32)	168 (90-245)	721 (454-997)	8553 (6684-10408)	9564 (7325-11799)	3.0 (2.3-3.7)	3.0 (2.3-3.8)
UR	2017/2018	114 (98-132)	23 (10-36)	189 (101-276)	822 (517-1135)	10024 (7833-12198)	11172 (8560-13777)	3.5 (2.7-4.3)	3.5 (2.7-4.3)
URC	2010/2011	131 (109-154)	14 (-1-30)	-228 (-412--36)	5147 (4505-5776)	16168 (11958-20246)	21232 (16158-26171)	6.9 (5.3-8.5)	8.0 (6.0-9.8)
URC	2011/2012	134 (111-157)	14 (-1-31)	-232 (-418--36)	5402 (4728-6062)	16842 (12456-21090)	22160 (16876-27304)	7.2 (5.5-8.8)	8.1 (6.2-10.0)
URC	2012/2013	138 (115-162)	15 (-1-32)	-247 (-446--39)	5638 (4935-6327)	18216 (13472-22810)	23760 (18075-29293)	7.6 (5.8-9.4)	8.4 (6.4-10.4)
URC	2013/2014	121 (100-142)	13 (-1-28)	-213 (-384--33)	4972 (4352-5580)	16490 (12196-20649)	21383 (16263-26365)	6.8 (5.2-8.4)	7.4 (5.6-9.1)
URC	2014/2015	125 (104-147)	13 (-1-28)	-223 (-402--35)	5178 (4533-5811)	17565 (12990-21995)	22659 (17224-27947)	7.2 (5.4-8.8)	7.6 (5.8-9.4)
URC	2015/2016	125 (104-147)	13 (-1-28)	-218 (-393--34)	5182 (4536-5816)	17982 (13299-22518)	23085 (17545-28475)	7.2 (5.5-8.9)	7.5 (5.7-9.3)
URC	2016/2017	123 (102-145)	13 (-1-28)	-218 (-394--34)	5182 (4536-5815)	18470 (13660-23128)	23570 (17903-29082)	7.3 (5.6-9.1)	7.5 (5.7-9.2)
URC	2017/2018	138 (114-162)	15 (-1-32)	-246 (-444--39)	5902 (5166-6623)	21646 (16009-27106)	27455 (20845-33885)	8.5 (6.5-10.5)	8.5 (6.5-10.5)
All Cause	2010/2011	140 (20-266)	185 (126-243)	-2011 (-2677--1318)	8551 (7456-9673)	24281 (18245-30604)	31146 (23170-39468)	10.1 (7.5-12.8)	11.7 (8.8-14.8)
All Cause	2011/2012	143 (21-272)	188 (128-246)	-2040 (-2716--1337)	8975 (7826-10152)	25293 (19005-31879)	32558 (24263-41212)	10.5 (7.8-13.3)	12.0 (8.9-15.1)
All Cause	2012/2013	147 (22-281)	193 (132-254)	-2176 (-2897--1426)	9368 (8168-10597)	27356 (20555-34480)	34889 (25980-44185)	11.2 (8.3-14.2)	12.4 (9.3-15.7)
All Cause	2013/2014	129 (19-246)	168 (115-220)	-1873 (-2493--1228)	8262 (7203-9345)	24764 (18607-31213)	31449 (23451-39796)	10.0 (7.5-12.7)	10.8 (8.1-13.7)
All Cause	2014/2015	133 (20-255)	173 (118-227)	-1961 (-2611--1285)	8604 (7502-9732)	26378 (19820-33247)	33327 (24849-42176)	10.5 (7.9-13.3)	11.2 (8.3-14.1)
All Cause	2015/2016	133 (19-254)	171 (117-225)	-1917 (-2552--1256)	8611 (7508-9740)	27006 (20292-34038)	34004 (25384-43001)	10.7 (8.0-13.5)	11.1 (8.3-14.0)
All Cause	2016/2017	131 (19-251)	171 (117-224)	-1921 (-2558--1259)	8610 (7507-9739)	27738 (20842-34961)	34728 (25927-43915)	10.8 (8.1-13.7)	11.0 (8.2-13.9)
All Cause	2017/2018	147 (21-280)	195 (133-255)	-2165 (-2882--1419)	9806 (8550-11092)	32508 (24426-40974)	40491 (30249-51183)	12.5 (9.4-15.9)	12.5 (9.4-15.9)

© 2022 Hansen CL et al. *JAMA Network Open.*

eTable 5. Estimated Excess Influenza Deaths and Mortality Rate per 100,000 Population by Season and Age Group, 1999/2000 to 2017/2018, USA

Cause of Death	Season	Deaths in <1 year (95% CI)	Deaths in 1 to 4 years (95% CI)	Deaths in 5 to 49 years (95%CI)	Deaths in 50 to 64 years (95% CI)	Deaths in 65+ years (95% CI)	Total influenza deaths (95% CI)	Influenza mortality rate per 100,000 population (95% CI)	Influenza mortality rate per 100,000 population, age-standardized to 2017/2018 population (95% CI)
UPI	1999/2000	16 (10-22)	14 (10-18)	380 (342-422)	506 (462-559)	7483 (7053-7972)	8398 (7876-8992)	3.0 (2.8-3.2)	3.8 (3.5-4.0)
UPI	2000/2001	32 (23-41)	-3 (-9-3)	146 (82-206)	103 (28-171)	69 (-507-682)	347 (-382-1103)	0.1 (-0.1-0.4)	0.1 (-0.2-0.5)
UPI	2001/2002	4 (-9-17)	14 (5-23)	136 (46-226)	406 (278-530)	5318 (4420-6203)	5878 (4740-6999)	2.1 (1.7-2.5)	2.6 (2.1-3.1)
UPI	2002/2003	34 (26-43)	38 (32-44)	57 (2-117)	21 (-53-106)	95 (-485-765)	245 (-478-1075)	0.1 (-0.2-0.4)	0.1 (-0.2-0.4)
UPI	2003/2004	59 (53-66)	49 (45-53)	364 (326-409)	480 (426-536)	6987 (6538-7472)	7939 (7388-8536)	2.8 (2.6-3.0)	3.4 (3.2-3.7)
UPI	2004/2005	10 (1-20)	21 (14-28)	249 (180-313)	288 (189-387)	5548 (4788-6199)	6116 (5171-6946)	2.1 (1.8-2.4)	2.6 (2.2-3.0)
UPI	2005/2006	32 (18-45)	-1 (-11-9)	85 (-15-185)	317 (170-453)	1952 (1032-2986)	2385 (1195-3678)	0.8 (0.4-1.3)	1.0 (0.5-1.5)
UPI	2006/2007	7 (-4-19)	18 (9-25)	142 (67-220)	227 (101-358)	1492 (678-2272)	1886 (851-2894)	0.6 (0.3-1.0)	0.8 (0.3-1.2)
UPI	2007/2008	10 (2-17)	36 (31-42)	354 (299-410)	585 (506-680)	4291 (3763-4827)	5276 (4601-5976)	1.8 (1.5-2.0)	2.1 (1.8-2.4)
UPI	2008/2009	26 (15-38)	32 (23-40)	244 (169-318)	230 (87-356)	701 (-139-1678)	1233 (155-2430)	0.4 (0.1-0.8)	0.5 (0.0-0.9)
UPI	2009/2010	30 (19-40)	44 (37-51)	1047 (990-1112)	899 (786-1012)	1297 (567-2076)	3316 (2399-4291)	1.1 (0.8-1.4)	1.2 (0.8-1.5)
UPI	2010/2011	6 (-5-17)	28 (20-36)	656 (582-735)	591 (466-722)	1125 (281-1906)	2405 (1343-3417)	0.8 (0.4-1.1)	0.8 (0.4-1.2)
UPI	2011/2012	-13 (-26--1)	-8 (-17-1)	9 (-71-88)	84 (-84-240)	1379 (399-2364)	1451 (201-2692)	0.5 (0.1-0.9)	0.5 (0.1-1.0)
UPI	2012/2013	14 (1-27)	27 (18-35)	337 (261-423)	648 (482-820)	9134 (8115-10223)	10158 (8877-11528)	3.3 (2.8-3.7)	3.7 (3.2-4.1)
UPI	2013/2014	10 (-1-21)	21 (13-30)	1553 (1480-1635)	2068 (1922-2220)	3239 (2205-4330)	6891 (5619-8237)	2.2 (1.8-2.6)	2.3 (1.9-2.8)
UPI	2014/2015	10 (-3-22)	13 (5-22)	482 (406-567)	659 (500-818)	10651 (9562-11778)	11816 (10471-13207)	3.7 (3.3-4.2)	4.0 (3.5-4.5)
UPI	2015/2016	16 (5-27)	32 (24-39)	522 (455-607)	1154 (1026-1310)	1521 (538-2553)	3246 (2049-4537)	1.0 (0.6-1.4)	1.0 (0.6-1.5)
UPI	2016/2017	21 (9-33)	4 (-4-12)	260 (177-341)	571 (411-718)	5067 (3906-6316)	5924 (4500-7420)	1.8 (1.4-2.3)	1.9 (1.4-2.4)
UPI	2017/2018	25 (14-35)	59 (51-66)	945 (877-1018)	2223 (2094-2371)	11839 (10803-12864)	15090 (13840-16354)	4.7 (4.3-5.1)	4.7 (4.3-5.1)
UR	1999/2000	44 (34-54)	20 (14-26)	553 (502-603)	1511 (1406-1622)	14809 (13968-15837)	16936 (15924-18142)	6.1 (5.7-6.5)	7.6 (7.2-8.1)
UR	2000/2001	51 (36-66)	10 (1-19)	64 (-10-146)	112 (-42-274)	156 (-1207-1587)	393 (-1223-2093)	0.1 (-0.4-0.7)	0.2 (-0.6-0.9)
UR	2001/2002	23 (1-46)	29 (14-45)	313 (206-428)	883 (615-1166)	11378 (9693-13372)	12626 (10529-15056)	4.5 (3.7-5.3)	5.6 (4.7-6.6)
UR	2002/2003	52 (36-67)	62 (52-72)	14 (-64-98)	168 (-6-338)	581 (-799-1800)	876 (-781-2376)	0.3 (-0.3-0.8)	0.4 (-0.3-1.0)
UR	2003/2004	65 (54-75)	61 (54-67)	482 (426-536)	1191 (1070-1326)	12821 (11980-13791)	14620 (13584-15793)	5.1 (4.7-5.5)	6.3 (5.9-6.8)
UR	2004/2005	34 (18-49)	14 (3-25)	410 (323-503)	1041 (844-1260)	12929 (11558-14327)	14428 (12745-16164)	5.0 (4.4-5.6)	6.2 (5.5-6.9)
UR	2005/2006	18 (-3-39)	0 (-15-16)	117 (-4-240)	601 (278-936)	5723 (3664-7850)	6458 (3919-9080)	2.2 (1.3-3.1)	2.7 (1.7-3.8)
UR	2006/2007	-32 (-50--13)	40 (26-53)	162 (67-262)	539 (256-793)	3756 (1965-5441)	4465 (2264-6537)	1.5 (0.8-2.2)	1.8 (0.9-2.7)
UR	2007/2008	5 (-8-19)	56 (47-65)	471 (402-540)	1421 (1239-1609)	10283 (9041-11406)	12236 (10721-13638)	4.1 (3.6-4.6)	5.0 (4.3-5.5)
UR	2008/2009	52 (32-70)	34 (21-47)	351 (263-463)	404 (128-689)	1745 (-45-3606)	2585 (399-4874)	0.9 (0.1-1.6)	1.0 (0.1-1.9)
UR	2009/2010	42 (26-59)	38 (26-48)	1253 (1165-1339)	1528 (1287-1781)	2424 (859-4111)	5284 (3363-7337)	1.7 (1.1-2.4)	1.9 (1.2-2.7)
UR	2010/2011	6 (-12-25)	10 (-2-23)	903 (804-996)	1361 (1051-1664)	2907 (1225-4724)	5187 (3066-7430)	1.7 (1.0-2.4)	1.9 (1.1-2.7)
UR	2011/2012	-28 (-48--7)	-21 (-36--8)	62 (-47-173)	408 (61-728)	3646 (1497-5803)	4068 (1426-6688)	1.3 (0.5-2.2)	1.5 (0.5-2.5)
UR	2012/2013	25 (5-46)	10 (-3-25)	493 (380-606)	2070 (1737-2433)	18510 (16151-20722)	21108 (18269-23833)	6.8 (5.9-7.6)	7.6 (6.6-8.5)
UR	2013/2014	3 (-17-21)	7 (-6-20)	1683 (1583-1787)	3204 (2886-3520)	6484 (4349-8740)	11380 (8795-14089)	3.6 (2.8-4.5)	3.8 (2.9-4.8)
UR	2014/2015	16 (-6-37)	5 (-9-18)	534 (423-654)	1473 (1137-1817)	17885 (15477-20216)	19914 (17023-22742)	6.3 (5.4-7.2)	6.7 (5.8-7.7)
UR	2015/2016	22 (5-42)	27 (15-39)	713 (620-807)	2234 (1935-2542)	3206 (787-5700)	6202 (3362-9130)	1.9 (1.1-2.9)	2.0 (1.1-2.9)
UR	2016/2017	16 (-3-36)	2 (-10-16)	288 (182-403)	1590 (1270-1911)	9410 (7005-11947)	11307 (8443-14313)	3.5 (2.6-4.5)	3.6 (2.7-4.6)
UR	2017/2018	16 (-2-33)	56 (43-67)	991 (899-1082)	3374 (3086-3656)	18739 (16616-21336)	23176 (20643-26174)	7.2 (6.4-8.1)	7.2 (6.4-8.1)

© 2022 Hansen CL et al. *JAMA Network Open.*

eTable 5. Estimated Excess Influenza Deaths and Mortality Rate per 100,000 Population by Season and Age Group, 1999/2000 to 2017/2018, USA, continued

Cause of Death	Season	Deaths in <1 year (95% CI)	Deaths in 1 to 4 years (95% CI)	Deaths in 5 to 49 years (95%CI)	Deaths in 50 to 64 years (95% CI)	Deaths in 65+ years (95% CI)	Total influenza deaths (95% CI)	Influenza mortality rate per 100,000 population (95% CI)	Influenza mortality rate per 100,000 population, age-standardized to 2017/2018 population (95% CI)
URC	1999/2000	50 (37-65)	45 (36-54)	999 (889-1125)	3763 (3533-4006)	35088 (33109-37512)	39945 (37604-42761)	14.4 (13.6-15.4)	18.0 (17.0-19.3)
URC	2000/2001	72 (51-93)	18 (6-30)	518 (333-700)	-267 (-665-125)	-3867 (-6860--842)	-3525 (-7136-106)	-1.3 (-2.5-0.0)	-1.7 (-3.3--0.1)
URC	2001/2002	56 (27-84)	29 (10-49)	349 (82-648)	1452 (793-2086)	23915 (19106-28457)	25801 (20018-31324)	9.1 (7.1-11.1)	11.4 (8.9-13.8)
URC	2002/2003	35 (15-55)	84 (71-97)	192 (10-389)	325 (-110-742)	-3312 (-6302--41)	-2676 (-6316-1242)	-0.9 (-2.2-0.4)	-1.2 (-2.8-0.5)
URC	2003/2004	67 (53-82)	62 (54-71)	838 (716-967)	3194 (2908-3483)	29833 (27837-32056)	33995 (31568-36659)	11.8 (11.0-12.7)	14.7 (13.7-15.8)
URC	2004/2005	10 (-11-33)	-2 (-17-12)	619 (407-821)	1621 (1074-2188)	25088 (21596-29048)	27334 (23048-32101)	9.4 (7.9-11.0)	11.7 (9.9-13.8)
URC	2005/2006	-21 (-55-13)	-11 (-31-12)	33 (-273-313)	91 (-695-845)	9182 (4141-14694)	9275 (3087-15877)	3.2 (1.1-5.4)	4.0 (1.4-6.7)
URC	2006/2007	-20 (-46-5)	33 (16-50)	336 (96-592)	1485 (848-2083)	8487 (4361-12930)	10320 (5276-15661)	3.5 (1.8-5.3)	4.3 (2.2-6.4)
URC	2007/2008	35 (17-54)	66 (55-78)	1078 (894-1252)	3205 (2764-3685)	20936 (18205-24187)	25321 (21935-29255)	8.5 (7.3-9.8)	10.2 (8.9-11.8)
URC	2008/2009	67 (40-97)	60 (43-79)	374 (136-625)	901 (200-1568)	67 (-4379-4305)	1468 (-3959-6673)	0.5 (-1.3-2.2)	0.5 (-1.6-2.5)
URC	2009/2010	5 (-19-27)	30 (15-46)	1277 (1085-1482)	2481 (1883-3044)	2304 (-1471-6558)	6097 (1493-11158)	2.0 (0.5-3.7)	2.2 (0.4-4.1)
URC	2010/2011	27 (5-52)	23 (7-40)	1216 (992-1425)	1127 (447-1807)	2692 (-1193-6932)	5086 (258-10255)	1.7 (0.1-3.3)	1.8 (-0.0-3.8)
URC	2011/2012	-18 (-48-12)	-15 (-35-5)	-314 (-555--48)	-120 (-840-642)	4266 (-1048-9526)	3799 (-2525-10137)	1.2 (-0.8-3.3)	1.5 (-0.9-3.8)
URC	2012/2013	-12 (-40-18)	0 (-21-19)	909 (642-1170)	3505 (2694-4343)	33822 (28122-38760)	38224 (31397-44310)	12.3 (10.1-14.2)	13.7 (11.3-15.9)
URC	2013/2014	6 (-22-31)	3 (-14-22)	2600 (2335-2827)	6199 (5466-6979)	9760 (4339-15046)	18568 (12105-24905)	5.9 (3.9-7.9)	6.2 (4.0-8.4)
URC	2014/2015	-9 (-36-18)	-4 (-24-16)	773 (524-1013)	2563 (1774-3375)	31715 (25715-37893)	35037 (27953-42316)	11.1 (8.8-13.4)	11.8 (9.5-14.3)
URC	2015/2016	25 (-3-51)	38 (22-54)	996 (776-1221)	3205 (2488-3870)	3256 (-1567-8756)	7520 (1715-13951)	2.4 (0.5-4.4)	2.4 (0.5-4.5)
URC	2016/2017	3 (-24-30)	1 (-17-16)	340 (67-582)	2365 (1592-3097)	12925 (6899-19112)	15634 (8517-22837)	4.9 (2.7-7.1)	5.0 (2.7-7.3)
URC	2017/2018	21 (-2-45)	65 (48-80)	1510 (1293-1746)	5937 (5252-6590)	29278 (24074-34648)	36812 (30665-43109)	11.4 (9.5-13.4)	11.4 (9.5-13.4)
All Cause	1999/2000	-45 (-117-25)	131 (101-164)	3084 (2664-3526)	5684 (5214-6120)	47406 (44393-50927)	56260 (52255-60761)	20.3 (18.9-21.9)	25.1 (23.4-27.1)
All Cause	2000/2001	103 (-6-199)	131 (85-180)	859 (260-1505)	-257 (-969-421)	-4683 (-9755-512)	-3847 (-10385-2818)	-1.4 (-3.7-1.0)	-1.9 (-4.7-1.0)
All Cause	2001/2002	-5 (-168-154)	85 (11-156)	2693 (1750-3623)	2145 (1146-3205)	35895 (28931-42923)	40812 (31669-50060)	14.4 (11.2-17.7)	17.8 (13.9-21.8)
All Cause	2002/2003	45 (-55-150)	22 (-20-71)	718 (67-1336)	97 (-675-786)	-5078 (-9774--428)	-4195 (-10457-1915)	-1.5 (-3.7-0.7)	-2.0 (-4.6-0.6)
All Cause	2003/2004	102 (26-179)	123 (92-156)	1954 (1547-2392)	4756 (4226-5284)	40067 (36664-43360)	47003 (42554-51372)	16.3 (14.8-17.8)	20.2 (18.3-22.0)
All Cause	2004/2005	272 (155-388)	11 (-46-68)	2325 (1609-3049)	2686 (1804-3565)	38822 (33715-43957)	44117 (37237-51028)	15.2 (12.8-17.5)	18.7 (15.9-21.6)
All Cause	2005/2006	73 (-81-231)	-9 (-87-67)	279 (-757-1373)	828 (-439-2084)	15665 (8233-23786)	16836 (6868-27541)	5.7 (2.3-9.4)	7.1 (3.1-11.5)
All Cause	2006/2007	66 (-69-213)	115 (50-175)	1239 (402-2066)	2756 (1541-3817)	13453 (7305-20070)	17630 (9228-26340)	5.9 (3.1-8.9)	7.2 (3.8-10.7)
All Cause	2007/2008	26 (-66-119)	116 (70-157)	2330 (1763-2907)	4815 (4077-5572)	33204 (29080-37539)	40491 (34924-46294)	13.5 (11.7-15.5)	16.3 (14.1-18.6)
All Cause	2008/2009	29 (-114-168)	79 (15-140)	1059 (212-1895)	1334 (277-2496)	-54 (-6516-6726)	2447 (-6127-11424)	0.8 (-2.0-3.8)	0.8 (-2.5-4.3)
All Cause	2009/2010	-13 (-128-106)	34 (-25-89)	1636 (955-2421)	3373 (2396-4318)	3228 (-2991-8991)	8257 (207-15925)	2.7 (0.1-5.2)	2.9 (-0.1-5.9)
All Cause	2010/2011	384 (257-501)	22 (-41-83)	2484 (1735-3280)	1872 (611-2973)	4737 (-1683-11141)	9499 (880-17979)	3.1 (0.3-5.9)	3.4 (0.2-6.5)
All Cause	2011/2012	94 (-47-244)	32 (-34-98)	-803 (-1696-140)	-59 (-1455-1291)	4609 (-3437-13092)	3873 (-6668-14864)	1.3 (-2.2-4.8)	1.5 (-2.3-5.5)
All Cause	2012/2013	-137 (-294-14)	-50 (-122-23)	3189 (2283-4178)	6364 (4892-7750)	54656 (45786-63517)	64023 (52545-75482)	20.5 (16.9-24.2)	22.9 (18.8-26.9)
All Cause	2013/2014	-53 (-187-90)	174 (101-239)	4199 (3251-5123)	9743 (8509-11078)	12645 (4979-20012)	26708 (16653-36542)	8.5 (5.3-11.6)	8.9 (5.4-12.3)
All Cause	2014/2015	113 (-42-251)	25 (-43-90)	1396 (491-2390)	4007 (2478-5405)	47956 (40286-56552)	53497 (43171-64688)	16.9 (13.7-20.5)	18.1 (14.6-21.8)
All Cause	2015/2016	251 (117-384)	-3 (-62-53)	2775 (2009-3635)	5103 (3884-6539)	5258 (-2369-13950)	13384 (3579-24561)	4.2 (1.1-7.7)	4.3 (1.1-7.9)
All Cause	2016/2017	34 (-105-173)	-127 (-193--64)	520 (-379-1402)	3073 (1689-4379)	18906 (10156-27542)	22405 (11167-33431)	7.0 (3.5-10.4)	7.1 (3.6-10.6)
All Cause	2017/2018	199 (79-313)	51 (-8-107)	2853 (2049-3608)	8997 (7865-10186)	44941 (36996-53787)	57041 (46981-68001)	17.7 (14.6-21.1)	17.7 (14.6-21.1)

© 2022 Hansen CL et al. *JAMA Network Open.*

eTable 6. Estimated Average Annual Age-Specific Influenza Deaths by Type/Subtype, 1999/2000 to 2017/2018, USA

Age in years	Cause of Death	A(H1) Deaths (95% CI)[a]	A(H3) Deaths (95% CI)	H1N1pdm09 Deaths (95% CI)[a]	B Deaths (95% CI)	Total deaths for all subtypes (95% CI)	Total deaths estimated by the final model (95% CI)
<1	UPI	5 (3-7)	12 (9-14)	4 (2-6)	-2 (-5-1)	18 (9-28)	18 (16-21)
	UR	4 (1-8)	16 (11-20)	6 (2-9)	-3 (-8-2)	22 (6-38)	23 (19-27)
	URC	8 (3-12)	14 (9-19)	4 (0-7)	-4 (-11-2)	21 (2-40)	21 (15-27)
1to4	UPI	2 (-0-4)	10 (8-13)	6 (4-8)	6 (2-9)	24 (14-34)	23 (21-25)
	UR	8 (5-11)	14 (10-17)	5 (2-7)	-2 (-6-3)	25 (12-38)	24 (21-27)
	URC	10 (6-14)	13 (9-18)	5 (2-8)	-0 (-6-5)	28 (11-46)	28 (24-31)
5to49	UPI	-38 (-75--1)	117 (72-163)	184 (153-216)	154 (100-209)	417 (249-586)	419 (403-436)
	UR	-48 (-85--10)	186 (140-232)	220 (188-252)	157 (103-212)	516 (346-686)	519 (497-541)
	URC	-31 (-104-43)	258 (168-348)	253 (190-315)	293 (185-401)	773 (439-1107)	771 (719-823)
50to64	UPI	-111 (-178--45)	131 (50-212)	185 (128-241)	424 (327-520)	628 (328-928)	635 (606-664)
	UR	-184 (-277--91)	470 (356-584)	295 (215-374)	728 (591-864)	1309 (885-1732)	1322 (1260-1384)
	URC	-140 (-359-78)	1202 (934-1470)	533 (347-720)	681 (361-1001)	2276 (1284-3268)	2265 (2120-2410)
65+	UPI	-331 (-627--35)	3631 (3267-3995)	115 (-138-368)	853 (419-1287)	4268 (2921-5615)	4168 (3968-4367)
	UR	-398 (-849-54)	7130 (6575-7684)	246 (-140-631)	1420 (759-2082)	8398 (6345-10450)	8284 (7855-8713)
	URC	-108 (-1035-819)	16206 (15069-17344)	209 (-582-1000)	-1557 (-2916--198)	14751 (10537-18965)	14496 (13465-15528)
Total	UPI	-473 (-877--70)	3901 (3406-4397)	494 (149-839)	1435 (843-2026)	5355 (3521-7191)	5263 (5014-5512)
	UR	-618 (-1205--28)	7816 (7092-8537)	772 (267-1273)	2300 (1439-3163)	10270 (7594-12944)	10171 (9652-10691)
	URC	-261 (-1489-966)	17693 (16189-19199)	1004 (-43-2050)	-587 (-2387-1211)	17849 (12273-23426)	17581 (16343-18819)

[a] Averages were calculated for full study period even for influenza A subtypes that did not circulate for the entire period.

© 2022 Hansen CL et al. *JAMA Network Open.*

eTable 7. Comparison With Other Published Estimates of Excess Influenza Mortality Rate for All Ages per 100,000 Population

Study	Years/Months Compared	Method	Cause of Death[a]	Estimate as published by authors[b]	Our estimates using different methodologies					
					(a) Estimate based on our final model	(b) All-ages Model	(c) No Spline	(d) No RSV	(e) Weekly influenza Type/Subtype Terms	(f) Multiple Causes instead of underlying cause of death
CDC/MMWR (2010)[10]	1999/2000 – 2006/2007	Regression	UPI	3.5 (3.3-3.6)	1.5 (1.5-1.6)	1.4 (1.3-1.5)	1.7 (1.7-1.8)	1.4 (1.4-1.5)	1.3 (1.3-1.4)	3.5 (3.4-3.7)
			URC	11.5 (11.0-11.9)	6.1 (5.9-6.5)	6.1 (5.8-6.5)	8.0 (7.7-8.4)	6.1 (5.9-6.5)	4.8 (4.7-5.1)	8.3 (8.0-8.8)
Goldstein, et al (2012)[2]	1999/2000 – 2006/2007	Regression	All-Cause	10.3 (9.5-11.1)	9.4 (9.0-10.0)	9.3 (8.9-9.9)	12.0 (11.6-12.4)	9.2 (8.8-9.8)	7.3 (7.1-7.7)	9.4 (9.0-10.0)
	1997/1998 – 2006/2007		UPI	1.7 (1.5-1.9)	1.5 (1.5-1.6)	1.4 (1.3-1.5)	1.7 (1.7-1.8)	1.4 (1.4-1.5)	1.3 (1.3-1.4)	3.5 (3.4-3.7)
			UR	3.6 (3.0-4.1)	3.1 (3.0-3.3)	2.9 (2.8-3.1)	3.2 (3.1-3.4)	3.1 (3.0-3.2)	2.5 (2.4-2.6)	5.8 (5.6-6.1)
			URC	8.2 (6.8-9.5)	6.1 (5.9-6.5)	6.1 (5.8-6.5)	8.0 (7.7-8.4)	6.1 (5.9-6.5)	4.8 (4.7-5.1)	8.3 (8.0-8.8)
Quandelacy, et al (2014) [1]	1999/2000 – 2006/2007	Regression	All-cause	10.1 (9.5-10.8)	9.4 (9.0-10.0)	9.3 (9.0-9.9)	12.0 (11.1-13.1)	9.2 (8.8-9.8)	7.3 (7.1-7.7)	9.4 (9.0-10.0)
Matias, et al (2014)[7]	1997/1998 – 04/2009	Regression	PI	3.7	1.4 (1.4-1.5)	1.3 (1.2-1.4)	1.6 (1.5-1.7)	1.4 (1.3-1.5)	1.3 (1.2-1.3)	3.3 (3.2-3.5)
			Resp	6.6	3.1 (3.0-3.3)	2.7 (2.6-2.9)	3.2 (3.1-3.4)	3.0 (2.9-3.2)	2.5 (2.4-2.6)	5.7 (5.4-6.0)
			RC	9.8	6.1(5.8-6.5)	5.6 (5.4-6.1)	7.1 (6.8-7.7)	5.9 (5.6-6.4)	4.6 (4.5-5.0)	8.2 (7.8-8.8)
			PI (RSV)	2.2	1.0 (1.0-1.1)	0.7 (0.7-0.8)	0.9 (0.8-0.9)		0.9 (0.9-0.9)	2.1 (2.0-2.2)
			Resp (RSV)	3.9	2.3 (2.2-2.3)	1.7 (1.6-1.8)	2.1 (2.0-2.2)		1.9 (1.8-2.0)	4.4 (4.2-4.5)
			RC (RSV)	6.0	5.7(5.5-5.9)	5.0 (4.8-5.2)	3.2 (2.8-3.4)		3.6 (3.3-3.7)	7.9 (7.5-8.1)
Shrestha, et all (2011)[11]	04/2009 – 04/2010	Multiplier	All-Cause	4.1 (2.9-6.0)	3.0 (-0.7-6.8)	3.4 (-0.4 – 7.2)	-4.0 (-8.2-0.7)	0.6 (-3.2-4.7)	4.2 (1.0-7.4)	3.0 (-0.7-6.8)
Simonsen, et al (2013)[12]	04/2009 – 12/2009	Regression	UR	2.8	2.1 (1.1-3.1)	2.0 (0.9-3.1)	1.9 (01.0-3.0)	1.5 (0.6-2.5)	2.6 (1.7-3.5)	3.2 (1.5-5.0)
Reed, et al (2015)[13]	2010/2011 – 2012/2013	Multiplier	Resp	4.9 (3.7-6.1)	3.3 (3.2-3.4)	3.4 (3.3-3.5)	2.4 (2.3-2.5)	3.7 (3.6-3.8)	3.7 (3.7-3.8)	5.8 (5.7-6.0)
Rolfes, et al (2018)[14]	2010/2011-2015/2016	Regression	PI	1.3-6.4[c]	1.9 (1.8-2.0)	1.9 (1.8-2.0)	1.4 (1.4-1.5)	2.0 (21.9-2.1)	1.7 (1.7-1.8)	3.4 (3.3-3.6)
	2010/2011-2013/2014		RC	3.9-18.0[c]	5.3 (5.1-5.6)	5.5 (5.3-5.8)	0.1 (-0.1-0.4)	6.1 (5.9-6.4)	5.0 (4.9-5.2)	7.7 (7.4-8.1)
CDC/Website[15]	2010/2011 – 2017/2018	Multiplier	All-Cause	12.0 (10.6-13.4)	9.9 (9.4-10.5)	10.9 (10.4-11.5)	7.8 (7.3-8.4)	10.8 (10.3-11.4)	10.0 (9.7-10.5)	9.9 (9.4-10.5)

[a] "U" indicates only underlying causes of death were considered. If no "U" indicated then contributing causes were also considered in the paper.

[b] Estimate presented in paper after converting to a rate per 100,000 population for all ages and only considering overlapping years where possible. Confidence intervals included where possible.

[c] Paper presented a range instead of a mean or individual seasons to calculate a mean.

© 2022 Hansen CL et al. *JAMA Network Open.*

eReferences.

1. Quandelacy TM, Viboud C, Charu V, Lipsitch M, Goldstein E. Age- and sex-related risk factors for influenza-associated mortality in the United States between 1997-2007. *Am J Epidemiol*. 2014;179(2). doi:10.1093/aje/kwt235
2. Goldstein E, Viboud C, Charu V, Lipsitch M. Improving the estimation of influenza-related mortality over a seasonal baseline. *Epidemiology*. 2012;23(6):829-838. doi:10.1097/EDE.0b013e31826c2dda
3. Muscatello DJ, Newall AT, Dwyer DE, MacIntyre CR. Mortality Attributable to Seasonal and Pandemic Influenza, Australia, 2003 to 2009, Using a Novel Time Series Smoothing Approach. *PLoS One*. 2013;8(6). doi:10.1371/journal.pone.0064734
4. Cohen C, Walaza S, Treurnicht FK, et al. In-A nd Out-of-hospital Mortality Associated with Seasonal and Pandemic Influenza and Respiratory Syncytial Virus in South Africa, 2009-2013. *Clin Infect Dis*. 2018;66(1). doi:10.1093/cid/cix740
5. Fox J, Weisberg S. an {R} Companion to Applied Regression, Third Edition. Published online 2019.
6. Thompson WW, Shay DK, Weintraub E, Cox N, Anderson LJ, Fukuda K. Mortality associated with influenza and respiratory syncytial virus in the United States. *J Am Med Assoc*. 2003;289(2):179-186. doi:10.1001/jama.289.2.179
7. Matias G, Taylor R, Haguinet F, Schuck-Paim C, Lustig R, Shinde V. Estimates of mortality attributable to influenza and RSV in the United States during 1997-2009 by influenza type or subtype, age, cause of death, and risk status. *Influenza Other Respi Viruses*. 2014;8(5):507-515. doi:10.1111/irv.12258
8. Bob Rudis. cdcfluview: Retrieve Flu Season Data from the United States Centers for Disease Control and Prevention ('CDC') "FluView" Portal. R Package version 0.9.4. Published 2021. Accessed March 2, 2021. https://cran.r-project.org/package=cdcfluview
9. Dushoff J, Plotkin JB, Viboud C, Earn DJD, Simonsen L. Mortality due to influenza in the United States - An annualized regression approach using multiple-cause mortality data. *Am J Epidemiol*. 2006;163(2). doi:10.1093/aje/kwj024
10. Thompson, MG, Shay, DK, Zhou H, Bridges, CB, Cheng PY, Burns E, Bresee JS, Cox NJ, Influenza Div, National Center for Immunization and Respiratory Diseases C. Estimates of deaths associated with Seasonal Influenza - United States, 1976-2007. *MMWR*. 2010;59(33).
11. Shrestha SS, Swerdlow DL, Borse RH, et al. Estimating the burden of 2009 pandemic influenza a (H1N1) in the United States (April 2009-April 2010). *Clin Infect Dis*. 2011;52(SUPPL. 1):75-82. doi:10.1093/cid/ciq012
12. Simonsen L, Spreeuwenberg P, Lustig R, et al. Global Mortality Estimates for the 2009 Influenza Pandemic from the GLaMOR Project: A Modeling Study. *PLOS Med*. 2013;10(11). doi:10.1371/journal.pmed.1001558
13. Reed C, Chaves SS, Daily Kirley P, et al. Estimating Influenza Disease Burden from Population-Based Surveillance Data in the United States. *PLoS One*. 2015;10(3). doi:10.1371/journal.pone.0118369
14. Rolfes MA, Foppa IM, Garg S, et al. Annual estimates of the burden of seasonal influenza in the United States: A tool for strengthening influenza surveillance and preparedness. *Influenza Other Respi Viruses*. 2018;12(1):132-137. doi:10.1111/irv.12486
15. Centers for Disease Control and Prevention. Past Seasons Estimated Influenza Disease Burden. https://www.cdc.gov/flu/about/burden/past-seasons.html

© 2022 Hansen CL et al. *JAMA Network Open.*
